# Anti-Cancer Stem Cell Properties of Square Planar Copper(II) Complexes with Vanillin Schiff Base Ligands

**DOI:** 10.3390/molecules30071636

**Published:** 2025-04-06

**Authors:** Yihan Wang, Kuldip Singh, Chunxin Lu, Kogularamanan Suntharalingam

**Affiliations:** 1 School of Chemistry, University of Leicester, Leicester LE1 7RH, UK; yw564@leicester.ac.uk (Y.W.); ks42@leicester.ac.uk (K.S.); 2 College of Biological, Chemical Sciences and Engineering, Jiaxing University, Jiaxing 314001, China

**Keywords:** metallopharmaceuticals, bioinorganic chemistry, copper, cancer stem cells, reactive oxygen species

## Abstract

Current breast cancer therapies are unable to positively impact the lives of a significant proportion of diagnosed patients (24% based on 10-year survival rate). Breast cancer relapse and metastasis, the leading cause of breast cancer-associated deaths, is linked to the existence of breast cancer stem cells (CSCs). Redox-modulating metal complexes have been used to perturb the redox balance in breast CSCs and effect cell death. Here, we sought to expand this promising class of anti-breast CSC agents. Specifically, we report the synthesis, and anti-breast CSC properties of a series of copper(II) complexes bearing regioisomeric vanillin Schiff base ligands (**1**–**4**). X-ray crystallography studies show that the copper(II) complexes **1**–**4** adopt square planar geometries with the copper(II) centre coordinated to two vanillin Schiff base ligands. The most effective copper(II) complex within the series **4** displays low micromolar potency towards breast CSCs, up to 4.6-fold higher than salinomycin and cisplatin. Mechanistic studies indicate that copper(II) complex **4** elevates reactive oxygen species levels in breast CSCs, leading to activation of the JNK/p38 pathway and caspase-dependent apoptosis. Overall, this work expands the library of anti-breast CSC copper(II) complexes and provides insight into their mode of action.

## 1. Introduction

According to the latest statistics, breast cancer accounted for 685,000 deaths worldwide [1]. In order to improve this outcome, new, improved, cost-effective therapies need to be developed. Existing breast cancer therapies are unable to benefit the lives of a significant proportion of diagnosed patients (24% of all breast cancer patients are expected to die 10 years post-diagnosis) [2]. Breast cancer recurrence and metastasis, the main reason for breast cancer-associated deaths, is strongly linked to the existence of breast cancer stem cells (CSCs), a sub-population of breast cancer cells that have the ability to self-renew, differentiate, and form secondary tumours [3,4]. The population of breast CSC-like cells in metastatic tumour(s) has been shown clinically to be higher than compared to the primary site(s), [5,6,7]. Breast CSCs are able to elude conventional chemotherapy and radiation regimens as they divide relatively slowly and these treatments tend to target fast-growing cells [8,9,10,11]. The very low proportion of breast CSCs within a given primary or metastatic tumour site and their tendency to reside in hard-to-reach niches means that they can be missed by surgery as well [12]. After surviving treatment, breast CSCs are believed to be able to regenerate tumours in the original site or produce invasive breast cancer cells that can colonise distant organs. The clinical implication of breast CSCs means that treatments must have the ability to remove heterogeneous breast cancer populations in their entirety, including breast CSCs, otherwise breast CSC-mediated relapse could occur. Potential breast CSC therapeutic targets such as cell surface markers, deregulated signalling pathways, and components within the microenvironments in which they reside have been identified, but there is still no clinically approved drug that can completely remove breast CSCs at their clinically administered dose(s) [13,14]. The current batch of small molecules undergoing investigation as breast CSC-specific agents are typically organic in nature [15,16]. We believe that the versatility of metal complexes, which arises from the choice of metal, oxidation state, redox activity, number and type of ligands, coordination geometry, magnetic and optical properties, deserves to be investigated further in the context of rational anti-breast CSC drug design [17].

The intracellular redox state of breast CSCs is finely controlled and balanced [18,19]. The tightly regulated redox environment in breast CSCs presents a potential therapeutic target. Reactive oxygen species (ROS)-generating metal complexes have been used to agitate the redox balance in breast CSCs to effect cell death [17,20,21,22]. We and others have shown that endogenous metal-based compounds (copper, iron, and manganese complexes) are able to exploit Fenton-type reactions and potently and selectively kill breast CSCs through redox stress-related mechanisms [23,24,25,26,27,28,29]. A number of mono-nuclear and multi-nuclear copper(II) complexes containing Schiff base ligands have been reported to kill breast CSCs (cultured in monolayers and as spheroids) in the micromolar range by elevating intracellular ROS levels above the lethal threshold [30,31,32,33]. In this study, we sought to expand the library of ROS-generating, breast CSC-potent copper(II) complexes by developing a new series of four-coordinate, square planar copper(II) complexes with regioisomeric vanillin Schiff base ligands. The synthesis, characterisation (including X-ray crystal structures), and in vitro anti-breast CSC activity of four copper(II) complexes **1**–**4** is reported, as well as the mechanism of action of the most effective copper(II) complex within the series.

## 2. Results and Discussion

**Figure 1:**
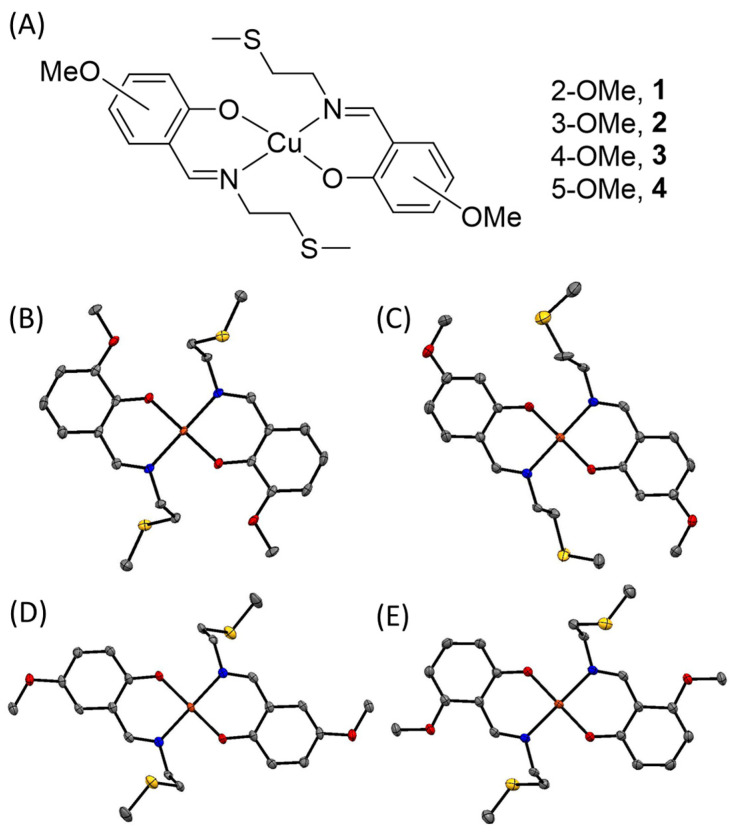
(**A**) Chemical structures of square planar copper(II) complexes **1**–**4** comprising of two vanillin Schiff base ligands. (**B**) X-ray structure of **1**. (**C**) X-ray structure of **2**. (**D**) X-ray structure of **3**. (**E**) X-ray structure of **4**. Thermal ellipsoids are drawn at 50% probability. C atoms are shown in grey, N in dark blue, O in red, S in yellow and Cu in orange. The H atoms have been omitted for clarity.

The vanillin Schiff base ligands (**L^1^**–**L^4^**, structures depicted in Figure S1) were prepared according to a previously reported protocol [30]. The corresponding copper(II) complexes **1**–**4** (Figure 1A) were prepared by reacting Cu(NO_3_)_2_⋅3H_2_O with 3.3- to 4-fold excess of the vanillin Schiff base ligands (**L^1^**–**L^4^**) in methanol. The copper(II) complexes **1**–**4** were isolated in reasonable to good yields (53–72%) as yellow or green solids, and characterised by high-resolution ESI mass spectrometry, infrared spectroscopy, and elemental analysis (Figures S2–S9, see Supplementary Materials). Distinctive molecular ion peaks corresponding to **1**–**4** with the appropriate isotopic pattern were observed in the high-resolution ESI mass spectra (*m*/*z* = 512.0865 a.m.u, [**1**+H]^+^; 512.0863 a.m.u. [**2**+H]^+^; 512.0856 a.m.u. [**3**+H]^+^; 512.0865 a.m.u. [**4**+H]^+^) (Figures S2–S5). The IR spectra of **1**–**4** displayed C=N imine stretching bands at relatively lower frequencies than the corresponding Schiff base ligands (**L^1^**–**L^4^**), further confirming the coordination of copper to **L^1^**–**L^4^** (Figures S6–S10). The purity of the solid form of **1**–**4** was confirmed by elemental analysis. Single crystals of **1**–**4** suitable for X-ray diffraction studies were obtained by slow diffusion of diethyl ether into an acetonitrile solution of **1**–**4** (CCDC 2426496–2426499, Figure 1B–E, Tables S1 and S2). Selected bond distances and angles data for **1**–**4** are presented in Tables S3–S6. The copper(II) complexes exhibit a distorted square planar geometry with the copper centre in **1**–**4** coordinated to two vanillin Schiff base ligands **L^1^**–**L^4^**, in head-to-tail arrangement, via the phenolate oxygen and the imine nitrogen atoms. The X-ray structure unambiguously shows that the thiol ether moiety does not coordinate with the copper(II) centre in **1**–**4**. Within the CuN_2_O_2_ plane, the average bond angles between adjacent atoms coordinated with the copper centre in **1**–**4** varied from 89.7° to 90.0°, consistent with a distorted square planar geometry. The average Cu-N_imine_ and Cu-O_phenolate_ bond lengths observed for **1**–**4** are consistent with related copper(II) complexes [31,32,34,35].

The lipophilicity of **1**–**4** was determined by measuring the extent to which it partitioned between octanol and water. The experimentally determined LogP values for **1**–**4** varied from 0.34 ± 0.003 to 0.75 ± 0.01 (Table S7). The relatively narrow range of LogP values determined for **1**–**4** suggests that the position of the methoxy group on the vanillin Schiff base ligands does not markedly influence lipophilicity. The LogP values for **1**–**4** indicate amphiphilicity and suggest that they should be sufficiently soluble in aqueous solutions to conduct cell-based studies and be readily taken up by dividing cells. Time course UV-vis spectroscopy and ESI mass spectrometry studies were conducted to evaluate structural integrity **1**–**4** in solutions relevant to biological studies. In DMSO (50 µM), the absorbance of the π-π* and MLCT bands associated with **1**–**4** remained largely unaltered over the course of 24 h at 37 °C, indicative of stability (Figure S11). This bodes well for the biological analysis of **1**–**4**, given that most agents are solubilised in DMSO prior to dilution in the appropriate cell media during biological studies. In PBS:DMSO (200:1) in the presence of ascorbic acid or glutathione (10 equivalents), cellular reductants, the UV-Vis trace for **1**–**4** (50 µM) changed dramatically over the course of 24 h at 37 °C (Figures S12 and S13). The spectral changes are consistent with partial ligand dissociation, as the final UV-vis trace of **1**–**4** was similar to the UV-Vis trace for the corresponding Schiff base ligands (**L^1^**–**L^4^**) (Figure S14). Upon addition of bathocuproine disulfonate (BCS, 2 equivalents), a strong copper(I) chelator, to a PBS:DMSO (200:1) solution of **1**–**4** (50 µM) and ascorbic acid or glutathione (10 equivalents), a characteristic absorbance band at 480 nm corresponding to [Cu^I^(BCS)_2_]^3−^ was observed in each case (Figures S15 and S16), suggestive of reduction of the copper(II) centre in **1**–**4** to copper(I) [36]. The ESI (positive) mass spectra of **1**–**4** (500 µM) in the presence of glutathione (10 equivalents) in H_2_O:DMSO (10:1) was dominated by a molecular ion peak corresponding to [**L^1^**+H]^+^, [**L^2^**+H]^+^, [**L^3^**+H]^+^ or [**L^4^**+H]^+^ (226.1 *m*/*z*) (Figure S17). Taken together, the UV-Vis spectroscopy and ESI spectrometry studies indicate that **1**–**4** are reduced from the copper(II) to copper(I) form under biologically reducing conditions and that this potentially leads to ligand dissociation and structural transformations at the copper centre.

The cytotoxicity of the copper(II) complexes **1**–**4** towards breast CSC-enriched (HMLER-shEcad) and breast CSC-depleted (HMLER) cells cultured in monolayers was determined using the colourimetric MTT (3-(4,5-dimethylthiazol-2-yl)-2,5-diphenyltetrazolium bromide) assay. The IC_50_ values associated to **1**–**4** were calculated from dose–response curves (Figures S18–S21) and are presented in Table 1. According to the IC_50_ values, **1**–**4** displayed low micromolar potency towards bulk breast cancer cells and breast CSCs. The IC_50_ values obtained for **1**–**4** varied across the series, but the variance was somewhat limited. This suggests that the position of the methoxy group on the vanillin Schiff base ligands present on **1**–**4** is not a major determinant of potency. Within the series, **4** exhibited the greatest potency towards bulk breast cancer cells and breast CSCs. Remarkably, **4** exhibited 3.4-fold (*p* < 0.05, n = 18) and 4.6-fold (*p* < 0.05, n = 18) greater potency towards breast CSCs than salinomycin and cisplatin, respectively (Table 1) [23,24]. Salinomycin is a polyether antibiotic and gold-standard anti-breast CSC agent, whereas cisplatin is the leading metallopharmaceutical used to treat various forms of cancer in the clinic [37,38]. Control studies showed that the IC_50_ value for **L^4^** was 3.8-fold (IC_50_ value = 4.13 ± 0.27 µM, *p* < 0.05, n = 18) and 6.1-fold (IC_50_ value = 7.56 ± 1.09 µM, *p* < 0.05, n = 18) greater than **4** towards HMLER cells and HMLER-shEcad cells, respectively (Figure S22). Further, Cu(NO_3_)_2_⋅3H_2_O was non-toxic towards HMLER and HMLER-shEcad cells (IC_50_ value > 100 µM in both cases) [39]. This suggests that the potency of **4** towards bulk breast cancer cells and breast CSCs can be attributed largely to the intact copper(II)-Schiff base complex rather than its individual constituents. To gauge the therapeutic potential of **1**–**4**, their cytotoxicity towards non-cancerous BEAS-2B (bronchial epithelium) and MCF10A (epithelial breast) cells was determined (Table S8, Figures S23 and S24). The copper(II) complexes **1**–**4** were significantly (up to 14-fold, *p* < 0.05, n = 18) less potent towards BEAS-2B and MCF10A cells than HMLER and HMLER-shEcad cells (Table S8, Figures S23 and S24). Therefore, **1**–**4** have the potential to selectively reduce the viability of bulk breast cancer cells and breast CSCs over non-cancerous lung and breast cells.

**Figure 2:**
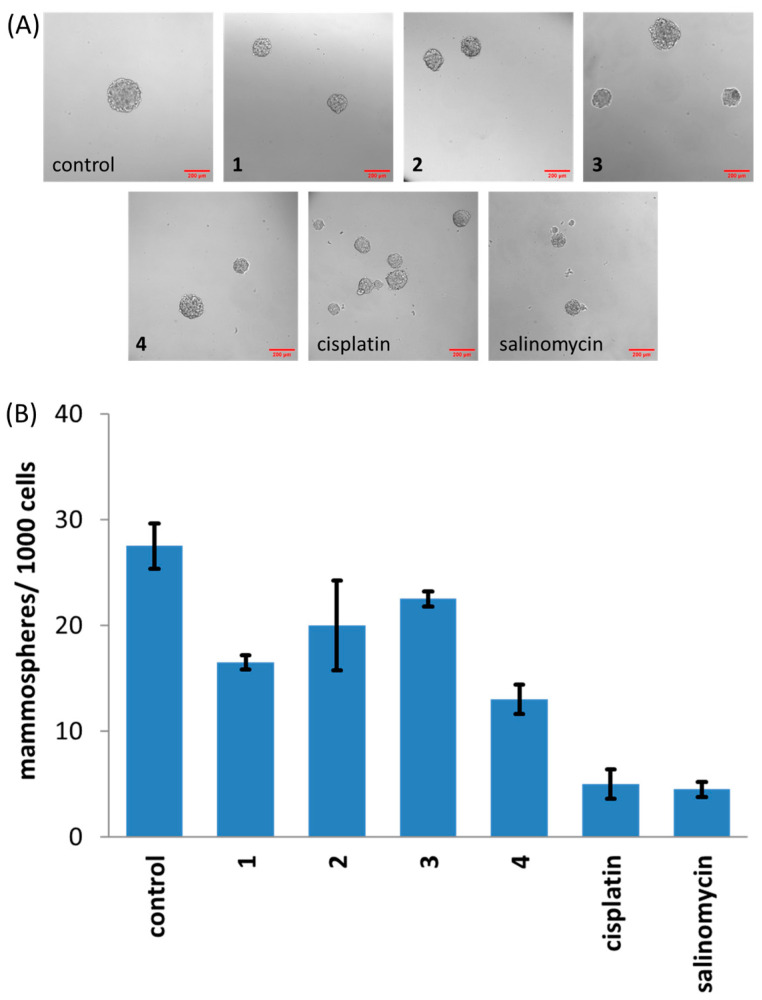
(**A**) Representative bright-field images (×10) of mammospheres in the absence and presence of copper(II) complexes **1**–**4**, cisplatin or salinomycin (IC_20_ value for 5 days). Scale bar = 200 µm. (**B**) Quantification of mammosphere formation with HMLER-shEcad cells untreated and treated with copper(II) complexes **1**–**4**, cisplatin or salinomycin (IC_20_ value for 5 days). Error bars represent standard deviations.

As the copper(II) complexes **1**–**4** were deemed to be significantly potent towards breast CSCs grown in two-dimensional cultures, we challenged them against breast CSC mammospheres grown in three-dimensional cultures. Mammospheres are widely accepted as a better representation of solid tumours than monolayers due to their three-dimensional architecture and the existence of an oxygen gradient within their structure [40,41,42]. The addition of **1**–**4** (IC_20_ value) to single cell suspensions of HMLER-shEcad cells, followed by incubation in low-attachment, serum-free conditions resulted in a reduction in the number and size of mammospheres formed compared to untreated HMLER-shEcad cells (Figure 2). The ability of **1**–**4** to reduce the size of mammospheres formed was comparable to salinomycin and cisplatin (under identical conditions); however, the ability of **1**–**4** to reduce the number of mammospheres formed was significantly lower than salinomycin and cisplatin (Figure 2). The colourimetric resazurin-based reagent, TOX8, was used to determine the ability of **1**–**4** to reduce mammosphere viability (Table 1, Figure S25). The potency of **1**–**4** towards mammospheres, based on the calculated IC_50_ values (concentration at which mammosphere viability is reduced by 50%), was in the micromolar range. As was observed for the monolayer-based cytotoxicity studies, **4** displayed the greatest potency towards mammospheres within the series. It should be noted that the potency of **1**–**4** towards mammospheres was lower than salinomycin and cisplatin (Table 1, Figure S25) [31,43].

**Table 1:** IC_50_ values of the copper(II) complexes **1**–**4**, cisplatin and salinomycin against HMLER and HMLER-shEcad cells and HMLER-shEcad mammospheres determined after 72 h or 120 h incubation (mean of two or three independent experiments ± SD).

Compound	HMLER IC_50_ [μM]	HMLER-shEcad IC_50_ [μM]	Mammosphere IC_50_ [μM]
1	3.13 ± 0.37	3.86 ± 0.54	35.85 ± 0.50
2	4.09 ± 0.06	4.26 ± 0.70	54.74 ± 3.08
3	2.35 ± 0.05	3.08 ± 0.08	31.78 ± 2.05
4	1.08 ± 0.06	1.24 ± 0.04	25.03 ± 1.89
cisplatin ^1^	2.57 ± 0.02	5.65 ± 0.30	13.50 ± 2.34
salinomycin ^1^	11.43 ± 0.42	4.23 ± 0.35	18.50 ± 1.50

^1^ Taken from references [23,24,30,43].

Additional cell-based studies were conducted to shed light on the mechanism of action of the copper(II) complexes. Cellular uptake studies were carried out to measure the whole cell uptake of **1**–**4** in breast CSCs (Figure S26). HMLER-shEcad cells treated with **1**–**4** (3 µM for 24 h) contained a relatively large amount copper (from 53.6 ± 0.3 ng of Cu/million cells for **4** to 77.2 ± 0.3 ng of Cu/million cells for **1**), suggesting that **1**–**4** are able to effectively penetrate and enter breast CSCs (Figure S26). Notably, the cellular uptake of the most potent copper(II) complex **4** was the lowest within the series. It is important to highlight that the IC_50_ values obtained for the copper(II) complexes **1**–**4** towards breast CSCs vary within a narrow window (Table 1). This is also true for the breast CSC uptake of the copper(II) complexes **1**–**4**. Therefore, the non-correlative relationship between breast CSC potency and uptake for the copper(II) complexes **1**–**4** is somewhat unsurprising. Fractionation studies with the most potent copper(II) complex within the series **4** showed that the vast majority of internalised **4** was detected in the cytoplasm with smaller but appreciable amounts of **4** detected in the nucleus and membrane (Figure S27). This implies that the mechanism of action of **4** involves cytoplasmic targets; however, nucleus or membrane-associated targets could not be completely ruled out.

**Figure 3:**
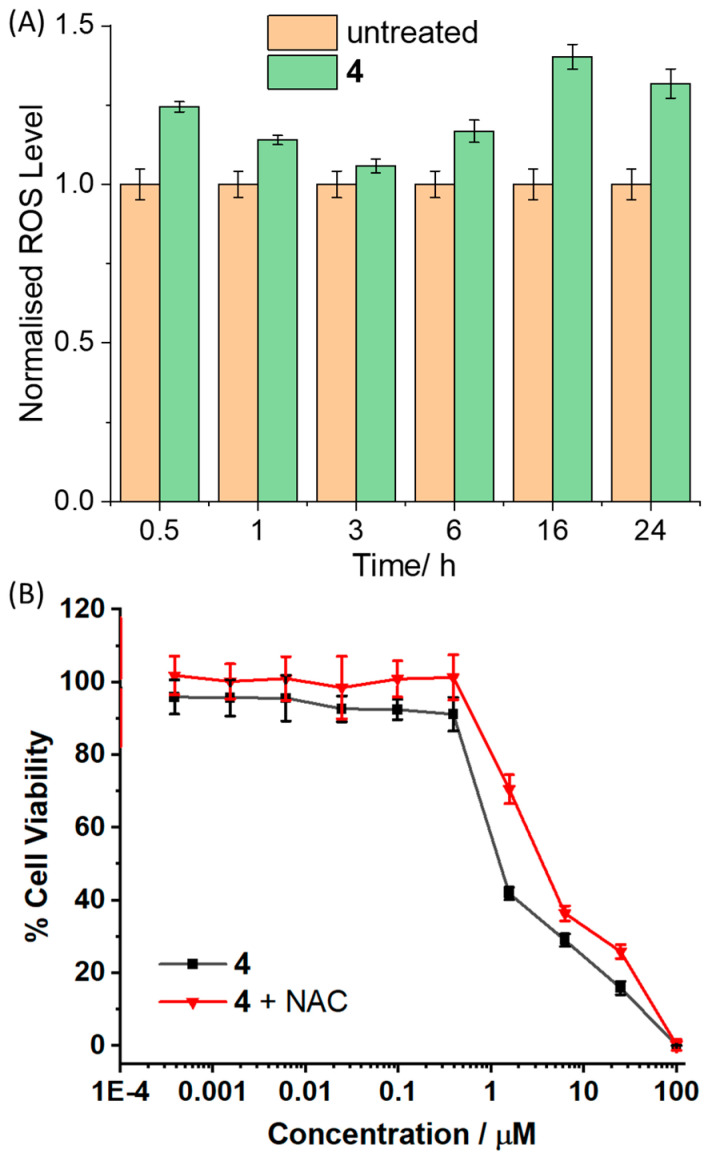
(**A**) Normalised ROS activity in untreated HMLER-shEcad cells (control) and HMLER-shEcad cells treated with **4** (IC_50_ value for 0.5, 1, 3, 6, 16, and 24 h). Error bars represent standard deviations. (**B**) Representative dose–response curves for the treatment of HMLER-shEcad cells with **4** after 72 h incubation in the presence and absence of *N*-acetylcysteine (2.5 mM).

The copper(II) complex **4** bearing two vanillin Schiff base ligands was envisaged to induce breast CSC toxicity by elevating intracellular ROS levels. A well-established ROS probe, 6-carboxy-2′,7′-dichlorodihydrofluorescein diacetate (DCFH-DA) was used to determine if **4** (IC_50_ value) could perturb intracellular ROS levels in breast CSCs over the course of 24 h (Figure 3A). The copper(II) complex **4** significantly increased intracellular ROS levels (*p* < 0.05) in breast CSCs upon short (0.5 h, 25% increase) and prolonged (16–24 h, 32–40% increase) exposure. Intracellular ROS production can activate stress pathways involving Jun-amino-terminal kinase (JNK) and/or p38 MAP kinase (MAPK) [44]. Immunoblotting studies showed that HMLER-shEcad cell treated with **4** (5–20 µM for 72 h) exhibited enhanced phosphorylation of JNK and p38 and their respective downstream effectors, c-Jun and MAP kinase-activated protein kinase 2 (MAPKAPK-2), respectively (Figure S28). This suggests that **4** induces JNK/p38 pathway activation in breast CSCs, most likely due to intracellular ROS elevation. Independent cytotoxicity studies in the presence of *N*-acetylcysteine (2.5 mM), a ROS scavenger, showed that the potency of **4** towards HMLER-shEcad cells significantly decreased (*p* < 0.05, IC_50_ value = 3.58 ± 0.30 µM, Figure 3B). This suggests that **4**-induced breast CSC death is related to intracellular ROS generation.

**Figure 4:**
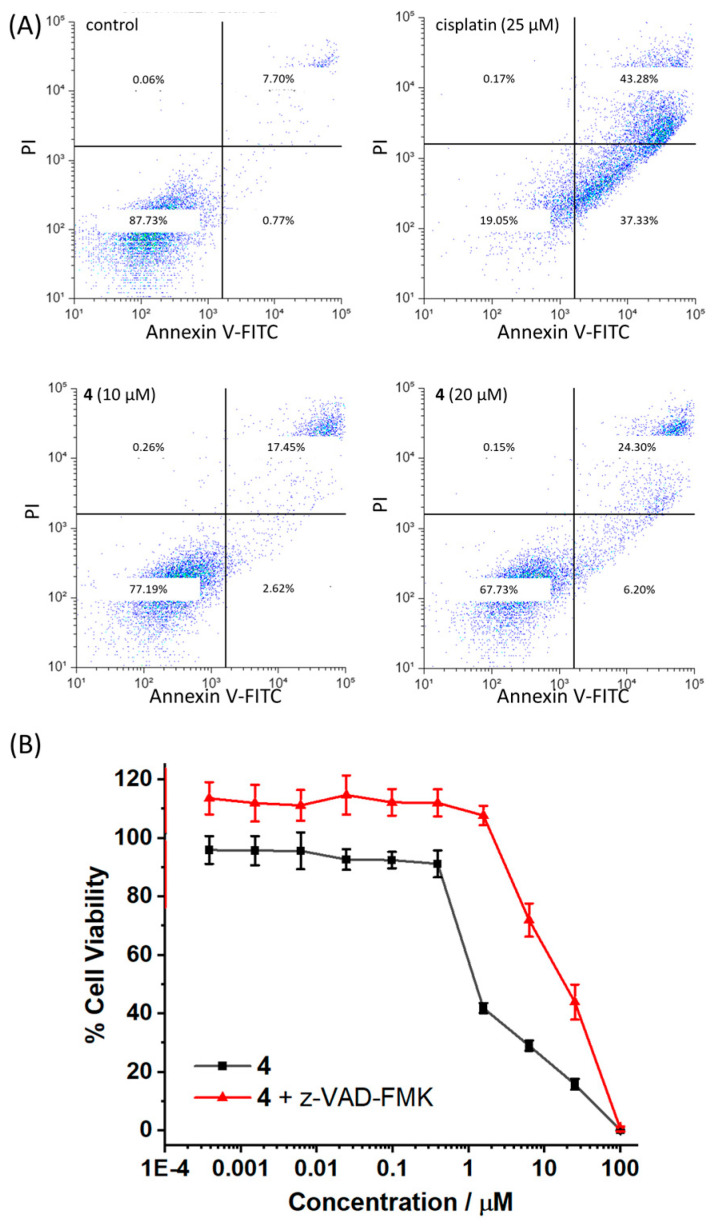
(**A**) FITC Annexin V-propidium iodide binding assay plots of untreated HMLER-shEcad cells and HMLER-shEcad cells treated with cisplatin (25 µM for 72 h), **4** (10 µM for 72 h) or **4** (20 µM for 72 h). (**B**) Representative dose–response curves for the treatment of HMLER-shEcad cells with **4** after 72 h incubation in the presence and absence of z-VAD-FMK (10 μM).

Elevation in intracellular ROS levels can overwhelm the cell’s inherent ROS buffering system and result in apoptotic cell death [45]. Apoptosis can lead to modification of the cell membrane architecture, including the presentation of phosphatidylserine residues on their exterior and an increase in permeability [46]. Phosphatidylserine residues can be readily sensed by Annexin V and the cell uptake of propidium iodide can provide insight with respect to cell membrane permeability [47]. The dual FITC Annexin V-propidium iodide staining flow cytometry assay was used to determine if **4** can induce morphological changes to breast CSCs consistent with apoptosis. The addition of **4** to HMLER-shEcad cells (10–20 µM for 72 h) induced a significant increase in the population of cells expressing late-stage apoptotic features (9.8–16.6% increase in late-stage apoptotic population, Figure 4A). A similar but more pronounced increase in the population of cells displaying apoptotic features was observed upon treatment of HMLER-shEcad cells with the well-established apoptosis inducer, cisplatin (25 µM for 72 h) (35.6% increase in late-stage apoptotic population, Figure 4A). Independent cytotoxicity studies involving the co-treatment of **4** with z-VAD-FMK (10 μM), a caspase-dependent apoptosis inhibitor [48], showed that the potency of **4** towards HMLER-shEcad cells significantly decreased (*p* < 0.05, IC_50_ value = 18.06 ± 0.78 µM, Figure 4B), suggesting that **4** induces caspase-dependent apoptotic breast CSC death. This was substantiated by immunoblotting studies of HMLER-shEcad cells treated with **4** (5–20 µM for 72 h) (Figure S28). The immunoblotting studies showed that there was a marked increase in the expression of cleaved caspase-3 and cleaved PARP-1 upon incubation of HMLER-shEcad cells with **4**. Overall, the mechanism of action studies suggests that **4** induces ROS elevation in breast CSCs, which leads to JNK/p38 pathway activation and ultimately caspase-dependent apoptosis.

## 3. Conclusions

In summary, we report the chemical preparation and full spectroscopic and analytical characterisation of four copper(II) complexes with regioisomeric vanillin Schiff base ligands (**1**–**4**). X-ray crystallography studies unambiguously indicate that the copper(II) complexes adopt square planar geometries with two vanillin Schiff base ligands in a head-to-tail arrangement. Interestingly, coordination of the copper(II) centre to the vanillin Schiff base ligands occurs via nitrogen and oxygen atoms and does not involve the thiol ether moiety. As expected, the copper(II) complexes **1**–**4** undergo reduction in the corresponding copper(I) form under biologically reducing conditions. Cytotoxicity studies showed that **1**–**4** display low micromolar potency towards bulk breast cancer cells and breast CSCs. The most potent copper(II) complex within the series **4** (containing the vanillin Schiff base ligand **L^4^**, with the methoxy group on the 5-position) displayed 3.4-fold and 4.6-fold greater potency towards breast CSCs than salinomycin and cisplatin, respectively. The anti-cancer potency of small molecules is multifaceted, and thus, from the data presented in this manuscript, it is difficult to pinpoint why **4** is the most potent copper(II) complex within the series; however, it is worth pointing out that the potency window for **1**–**4** towards bulk breast cancer cells and breast CSCs is relatively narrow (1.08–4.09 µM for HMLER cells and 1.24–4.26 µM for HMLER-shEcad cells). The copper(II) complexes **1**–**4** were significantly more (up to 14-fold) potent towards bulk breast cancer cells and breast CSCs than non-cancerous lung and breast cells. The copper(II) complex **4** was able to readily enter breast CSCs and largely localise in the cytoplasm. Mode of action studies indicated that **4** was able to significantly elevate intracellular ROS levels upon short and long exposure times, activate the JNK/p38 stress pathways (evidenced by the increase in expression of the phosphorylated forms of JNK, p38, c-Jun, and MAPKAPK-2), and evoke morphological changes consistent with caspase-dependent apoptosis. Overall, we report a novel series of ROS-modulating copper(II) complexes with promising anti-breast CSC activity and show that the most effective complex within the series exhibits a distinctive mode of action. Our results reinforce the biological potential of coordination copper complexes and moreover show that they deserve further attention as anti-breast CSC agents. Naturally, the next step will be to study the lead copper(II) complex **4** presented here, in in vivo systems. Future studies will focus on initially determining the safety profile of **4** in mice followed by tumour growth inhibition studies. If tumour growth is significantly inhibited, biodistrubution and histology studies will be warranted.

## 4. Materials and Methods

### 4.1. General Procedures

All synthetic procedures were performed under normal atmospheric conditions. Fourier transform infrared (FTIR) spectra were recorded with an IRAffinity-1S Shimadzu spectrophotometer. UV-Vis absorption spectra were recorded on a Cary 3500 UV-Vis spectrophotometer. Inductively coupled plasma mass spectrometry (ICP-MS) were measured using a Thermo Scientific ICAP-Qc quadrupole ICP mass spectrometer. Elemental analysis of the compounds prepared was performed commercially by the University of Cambridge. The vanillin Schiff base ligands **L^1^**–**L^4^** were prepared using a reported protocol [30]. Cu(NO_3_)_2_⋅3H_2_O was purchased from Sigma-Aldrich (St. Louis, MO, USA) and used without further purification. Solvents were purchased from Fisher and used without further purification.

### 4.2. Synthesis of Cu(***L^1^***)_2_ (***1***)

To a 10 mL methanolic solution of **L^1^** (113 mg, 0.50 mmol) was added a 10 mL methanolic solution of Cu(NO_3_)_2_⋅3H_2_O (31 mg, 0.13 mmol) in a dropwise manner. The solution was then stirred for 0.5 h. A yellow precipitate formed, which was collected by filtration. The collected solid was washed with methanol (10 mL) and diethyl ether (10 mL), and dried to give **1** as a yellow solid (35.5 mg, 53 %); ATR-FTIR (solid, cm^−1^): 2998, 2916, 2825, 1623, 1600, 1549, 1475, 1452, 1434, 1397, 1337, 1241, 1227, 1190, 1167, 1112, 1080, 1043, 974, 863, 730, 657, 625, 565, 514, 464, 435; HR ESI-MS: Calcd. for C_22_H_29_CuN_2_O_4_S_2_ [M + H]^+^ 512.0865 a.m.u. Found [M + H]^+^ 512.0865 a.m.u.; Anal. Calcd. for C_22_H_28_CuN_2_O_4_S_2_ (%): C 51.60, H 5.51, N 5.47. Found: C 51.48, H 5.44, N 5.51.

### 4.3. Synthesis of Cu(***L^2^***)_2_ (***2***)

To a 10 mL methanolic solution of **L^2^** (226 mg, 1.00 mmol) was added a 10 mL methanolic solution of Cu(NO_3_)_2_⋅3H_2_O (61 mg, 0.25 mmol) in a dropwise manner. The solution was then and stirred for 24 h. The solution mixture was concentrated by evaporation to ca. 5 mL, resulting in the formation of green crystals, which were filtered and washed with methanol (10 mL) and diethyl ether (10 mL). The solid was dried to give **2** as a green solid (90 mg, 70 %); ATR-FTIR (solid, cm^−1^): 2969, 2911, 1617, 1602, 1532, 1497, 1442, 1432, 1392, 1367, 1316, 1272, 1226, 1206, 1171, 1121, 1026, 976, 921, 826, 786, 746, 656, 611, 581, 516, 466, 451, 411; HR ESI-MS: Calcd. for C_22_H_29_CuN_2_O_4_S_2_ [M + H]^+^ 512.0865 a.m.u. Found [M + H]^+^ 512.0863 a.m.u.; Anal. Calcd. for C_22_H_28_CuN_2_O_4_S_2_ (%): C 51.60, H 5.51, N 5.47. Found: C 51.39, H 5.42, N 5.46.

### 4.4. Synthesis of Cu(***L^3^***)_2_ (***3***)

To a 10 mL methanolic solution of **L^3^** (113 mg, 0.50 mmol) was added a 10 mL methanolic solution of Cu(NO_3_)_2_⋅3H_2_O (31 mg, 0.13 mmol) in a dropwise manner. The solution was then stirred for 24 h. A yellow precipitate formed, which was collected by filtration. The collected solid was washed with methanol (10 mL) and diethyl ether (10 mL), and dried to give **3** as a yellow solid (46 mg, 72%); ATR-FTIR (solid, cm^−1^): 2993, 2916, 1624, 1610, 1546, 1481, 1444, 1426, 1398, 1366, 1320, 1287, 1255, 1218, 1200, 1163, 1117, 1038, 978, 845, 817, 766, 656, 575, 523, 490, 458, 380; HR ESI-MS: Calcd. for C_22_H_29_CuN_2_O_4_S_2_ [M + H]^+^ 512.0865 a.m.u. Found [M + H]^+^ 512.0856 a.m.u.; Anal. Calcd. for C_22_H_28_CuN_2_O_4_S_2_ (%): C 51.60, H 5.51, N 5.47. Found: C 51.60, H 5.44, N 5.49.

### 4.5. Synthesis of Cu(***L^4^***)_2_ (***4***)

To a 10 mL methanolic solution of **L^4^** (226 mg, 1.00 mmol) was added a 10 mL methanolic solution of Cu(NO_3_)_2_⋅3H_2_O (72 mg, 0.30 mmol) in a dropwise manner. The solution was then stirred for 24 h. A yellow precipitate formed, which was collected by filtration. The collected solid was washed with methanol (10 mL) and diethyl ether (10 mL), and dried to give **4** as a yellow solid (68 mg, 55%); ATR-FTIR (solid, cm^−1^): 2967, 2919, 1612, 1544, 1466, 1439, 1393, 1361, 1325, 1252, 1193, 1101, 1047, 1038, 928, 846, 787, 727, 654, 618, 549, 499, 476, 426; HR ESI-MS: Calcd. for C_22_H_29_CuN_2_O_4_S_2_ [M + H]^+^ 512.0865 a.m.u. Found [M + H]^+^ 512.0865 a.m.u.; Anal. Calcd. for C_22_H_28_CuN_2_O_4_S_2_ (%): C 51.60, H 5.51, N 5.47. Found: C 51.89, H 5.52, N 5.51.

### 4.6. X-Ray Crystallography

Crystals were mounted in inert oil on glass fibres and transferred to a Bruker Apex 2000 CCD area detector diffractometer. Data were collected using graphite-monochromated Mo-Kα radiation (λ = 0.71073) at 150(2) K. Scan type ϖ. Absorption corrections based on multiple scans were applied using SADABS [49] or spherical harmonics implemented in SCALE3 ABSPACK scaling algorithm [50]. The structures were solved by direct methods and refined on *F^2^* using the program SHELXT-2016 [51]. All non-hydrogen atoms were refined anisotropically. The CCDC deposition numbers 2426496–2426499 contain the supplementary crystallographic data. These data can be obtained free of charge via The Cambridge Crystallography Data Centre.

### 4.7. Measurement of Water-Octanol Partition Coefficient (LogP)

The LogP value for **1**–**4** was determined using the shake-flask method and UV-vis spectroscopy. The 1-octanol used in this experiment was pre-saturated with water. A DMSO solution of **1**–**4** (10 μL, 10 mM) was incubated with 1-octanol (495 μL) and H_2_O (495 μL) in a 1.5 mL tube. The tube was shaken at room temperature for 24 h. The two phases were separated by centrifugation and the content of **1**–**4** in the water and 1-octanol phases was determined by UV-vis spectroscopy.

### 4.8. Cell Culture

The human mammary epithelial cell lines, HMLER and HMLER-shEcad were kindly donated by Prof. R. A. Weinberg (Whitehead Institute, MIT). The human epithelial breast MCF10A cell line was acquired from American Type Culture Collection (ATCC, Manassas, VA, USA). HMLER, HMLER-shEcad, and MCF10A cells were maintained in Mammary Epithelial Cell Growth Medium (MEGM) with supplements and growth factors (BPE, hydrocortisone, hEGF, insulin, and gentamicin/amphotericin-B). The BEAS-2B bronchial epithelium cell line was acquired from American Type Culture Collection (ATCC, Manassas, VA, USA) and cultured in RPMI 1640 medium with 2 mM L-glutamine supplemented with 1% penicillin and 10% fetal bovine serum. The cells were grown at 310 K in a humidified atmosphere containing 5% CO_2_.

### 4.9. Cytotoxicity Studies: MTT Assay

Exponentially growing cells were seeded at a density of approximately 5 × 10^3^ cells per well in 96-well flat-bottomed microplates and allowed to attach for 24 h prior to addition of compounds. Various concentrations of the test compounds (0.0004–100 μM) were added and incubated for 72 h at 37 °C (total volume 200 μL). Stock solutions of the compounds were prepared as 10 mM DMSO solutions and diluted using cell media. The final concentration of DMSO in each well was ≤1 %. After 72 h, 20 μL of MTT (4 mg mL^−1^ in PBS) was added to each well and the plates incubated for an additional 4 h at 37 °C. The media/MTT mixture was eliminated and DMSO (100 μL per well) was added to dissolve the formazan precipitates. The optical density was measured at 550 nm using a 96-well multiscanner autoreader. Absorbance values were normalised to (DMSO-containing) control wells and plotted as the concentration of compound versus % cell viability. IC_50_ values were interpolated from the resulting dose-dependent curves. The reported IC_50_ values are the average of three independent experiments, each consisting of six replicates per concentration level (overall n = 18).

### 4.10. Tumorsphere Formation and Viability Assay

HMLER-shEcad cells (5 × 10^3^) were plated in ultralow-attachment 96-well plates (Corning, New York, NY, USA) and incubated in MEGM supplemented with B27 (Invitrogen, Carlsbad, CA, USA), 20 ng mL^−1^ EGF and 4 μg mL^−1^ heparin (Sigma, Livonia, MI, USA) for 5 days. Studies were also conducted in the presence of **1**–**4**, cisplatin, and salinomycin (0–133 µM). Mammospheres treated with **1**–**4**, cisplatin, and salinomycin (at their IC_20_ value, 5 days) were manually counted and imaged using an inverted microscope. The viability of the mammospheres was determined by the addition of a resazurin-based reagent, TOX8 (Sigma). After incubation for 16 h, the fluorescence of the solutions was read at 590 nm (λ_ex_ = 560 nm). Viable mammospheres reduce the amount of the oxidised TOX8 form (blue) and concurrently increase the amount of the fluorescent TOX8 intermediate (red), indicating the degree of mammosphere cytotoxicity caused by the test compound. Fluorescence values were normalised to DMSO-containing controls and plotted as the concentration of test compound versus % mammospheres viability. IC_50_ values were interpolated from the resulting dose-dependent curves. The reported IC_50_ values are the average of two independent experiments, each consisting of two replicates per concentration level (overall n = 4).

### 4.11. Cellular Uptake

To measure the cellular uptake of **1**–**4**, about 1 million HMLER-shEcad cells were treated with **1**–**4** (3 μM) at 37 °C for 24 h. After incubation, the media was removed, the cells were washed with PBS (2 mL × 3) and harvested. The number of cells was counted at this stage, using a haemocytometer. This mitigates any cell death induced by **1**–**4** at the administered concentration and experimental cell loss. The cellular pellets were dissolved in 65% HNO_3_ (250 µL) overnight. For **4**, the pellet was also used to determine the copper content in the cytoplasmic, nuclear, and membrane fractions. The Thermo Scientific NE-PER Nuclear and Cytoplasmic Extraction Kit was used to extract and separate the cytoplasmic, nuclear, and membrane fractions. The fractions were dissolved in 65% HNO_3_ (250 µL final volume) overnight. All samples were diluted 17-fold with water and analysed using inductively coupled plasma mass spectrometry (ICP-MS, Thermo Scientific ICAP-Qc quadrupole ICP mass spectrometer). Copper levels are expressed as mass of Cu (ng) per million cells. Results are presented as the mean of three determinations for each data point.

### 4.12. Intracellular ROS Assay

HMLER-shEcad cells (5 × 10^3^) were seeded in each well of a 96-well plate. After incubating the cells overnight, they were treated with **4** (IC_50_ value for 0.5–24 h), and incubated with 6-carboxy-2′,7′-dichlorodihydrofluorescein diacetate (20 μM) for 30 min. The intracellular ROS level was determined by measuring the fluorescence of the solutions in each well at 529 nm (λ_ex_ = 504 nm).

### 4.13. Immunoblotting Analysis

HMLER-shEcad cells (5 × 10^6^) were incubated with **4** (5–20 µM for 72 h) at 37 °C. HMLER-shEcad cells were harvested and isolated as pellets. SDS-PAGE loading buffer (64 mM Tris-HCl (pH 6.8), 9.6% glycerol, 2% SDS, 5% β-mercaptoethanol, 0.01% bromophenol blue) was added to the pellets and this was incubated at 95 °C for 10 min. Lysates were resolved by 4–20% sodium dodecylsulphate polyacylamide gel electrophoresis (SDS-PAGE; 200 V for 25 min) followed by electro transfer to polyvinylidene difluoride membrane, PVDF (350 mA for 1 h). Membranes were blocked in 5% (*w*/*v*) non-fat milk in PBST (PBS/0.1% Tween 20) and incubated with the appropriate primary antibodies (Cell Signalling Technology, Danvers, MA, USA). After incubation with horseradish peroxidase-conjugated secondary antibodies (Cell Signalling Technology, Danvers, MA, USA), immune complexes were detected with the ECL detection reagent (BioRad, Hercules, CA, USA) and analysed using a chemiluminescence imager (Bio-Rad ChemiDoc Imaging System).

### 4.14. Annexin V-Propidium Iodide Assay

HMLER-shEcad cells were incubated with and without **4** (10–20 µM for 72 h) and cisplatin (25 µM for 72 h) at 37 °C. Cells were harvested from adherent cultures by trypsinisation. The FITC Annexin V/Dead Cell Apoptosis Kit was used. The manufacture’s (Thermo Fisher Scientific, Waltham, MA, USA) protocol was followed to carry out this experiment. Briefly, untreated and treated cells (1 × 10^6^) were suspended in 1× Annexin binding buffer (100 µL) (10 mM HEPES, 140 mM NaCl, 2.5 mM CaCl_2_, pH 7.4), then 5 µL of FITC Annexin V and 1 µL of PI (100 µg/mL) were added to each sample and incubated at room temperature for 15 min. After which more 1× Annexin binding buffer (400 µL) was added while gently mixing. The cells were analysed using a FACSCanto II flow cytometer (BD Biosciences, Milpitas, CA, USA) (10,000 events per sample were acquired) at the University of Leicester FACS Facility. The FL1 channel was used to assess Annexin V binding and the FL2 channel was used to assess PI uptake. Cell populations were analysed using Floreada.io.

## Data Availability

Samples of the compounds are available from the authors.

## Supplementary Materials

The following supporting information can be downloaded at: https://www.mdpi.com/article/10.3390/molecules30071636/s1.
