# Supplementary material for: Anti-Cancer Stem Cell Properties of Square Planar Copper(II) Complexes with Vanillin Schiff Base Ligands
Source: Molecules. 2025 Apr 6;30(7):1636. doi: 10.3390/molecules30071636 (PMC11990672; doi:10.3390/molecules30071636)
Supplement: Supplementary file 1 [file molecules-30-01636-s001.zip › molecules-3531694-supplementary.pdf]

Supporting Information for

Anti-cancer stem cell properties of square planar copper(II) complexes with vanillin Schiff base ligands

Yihan Wang [1], Kuldip Singh [1], Chunxin Lu [2,*] and Kogularamanan Suntharalingam [1,*]

[1] School of Chemistry, University of Leicester, Leicester LE1 7RH, UK
[2] College of Biological, Chemical Sciences and Engineering, Jiaxing University, Jiaxing 314001, China

* To whom correspondence should be addressed:
Email: k.suntharalingam@leicester.ac.uk; chunxin.lu@zjxu.edu.cn

Table of Content

Figure S1. Chemical structures of the vanillin Schiff base ligands **L^1-L^4**.
Figure S2. High resolution ESI mass spectrum (positive mode) of **1**.
Figure S3. High resolution ESI mass spectrum (positive mode) of **2**.
Figure S4. High resolution ESI mass spectrum (positive mode) of **3**.
Figure S5. High resolution ESI mass spectrum (positive mode) of **4**.
Figure S6. ATR-FTIR spectrum of **1** in the solid form.
Figure S7. ATR-FTIR spectrum of **2** in the solid form.
Figure S8. ATR-FTIR spectrum of **3** in the solid form.
Figure S9. ATR-FTIR spectrum of **4** in the solid form.
Figure S10. ATR-FTIR spectrum of (A) **L^1**, (B) **L^2**, (C) **L^3**, and (D) **L^4** in the liquid form.
Table S1. Crystallographic data for copper(II) complexes **1** and **2**.
Table S2. Crystallographic data for copper(II) complexes **3** and **4**.
Table S3. Selected bond lengths (Å) and angles (°) for copper(II) complex **1**.
Table S4. Selected bond lengths (Å) and angles (°) for copper(II) complex **2**.
Table S5. Selected bond lengths (Å) and angles (°) for copper(II) complex **3**.
Table S6. Selected bond lengths (Å) and angles (°) for copper(II) complex **4**.
Table S7. Experimentally determined LogP values for the copper(II) complexes **1-4**.

Figure S11. UV-Vis spectra of (A) **1**, (B) **2**, (C) **3**, and (D) **4** (all 50 μM) in DMSO over the course of 24 h at 37 ^{o}C.

Figure S12. UV-Vis spectra of (A) **1**, (B) **2**, (C) **3**, and (D) **4** (all 50 μM) in PBS:DMSO (200:1) in the presence of ascorbic acid (10 equivalences) over the course of 24 h at 37 ^{o}C.

Figure S13. UV-Vis spectra of (A) **1**, (B) **2**, (C) **3**, and (D) **4** (all 50 μM) in PBS:DMSO (200:1) in the presence of glutathione (10 equivalences) over the course of 24 h at 37 ^{o}C.

Figure S14. UV-Vis spectra of $\mathbf{L^1}$, $\mathbf{L^2}$, $\mathbf{L^3}$, and $\mathbf{L^4}$ (all 50 μM) in PBS:DMSO (200:1) at 37 ^{o}C.

Figure S15. UV-Vis spectra of (A) **1**, (B) **2**, (C) **3**, and (D) **4** (all 50 μM) in PBS:DMSO (200:1) in the presence of ascorbic acid (10 equivalences) and bathocuproine disulfonate, BCS (2 equivalences) at 37 ^{o}C.

Figure S16. UV-Vis spectra of (A) **1**, (B) **2**, (C) **3**, and (D) **4** (all 50 μM) in PBS:DMSO (200:1) in the presence of glutathione (10 equivalences) and bathocuproine disulfonate, BCS (2 equivalences) at 37 ^{o}C.

Figure S17. ESI mass spectra (positive mode) of (A) **1**, (B) **2**, (C) **3**, and (D) **4** (all 500 μM) in H_2O:DMSO (10:1) in the presence of glutathione (10 equivalences) after incubation for 24 h at 37 ^{o}C.

Figure S18. Representative dose-response curves for the treatment of (A) HMLER and (B) HMLER-shEcad cells with **1** after 72 h incubation.

Figure S19. Representative dose-response curves for the treatment of (A) HMLER and (B) HMLER-shEcad cells with **2** after 72 h incubation.

Figure S20. Representative dose-response curves for the treatment of (A) HMLER and (B) HMLER-shEcad cells with **3** after 72 h incubation.

Figure S21. Representative dose-response curves for the treatment of (A) HMLER and (B) HMLER-shEcad cells with **4** after 72 h incubation.

Figure S22. Representative dose-response curves for the treatment of (A) HMLER and (B) HMLER-shEcad cells with $\mathbf{L^4}$ after 72 h incubation.

Figure S23. Representative dose-response curves for the treatment of BEAS-2B cells with (A) **1**, (B) **2**, (C) **3** or (D) **4** after 72 h incubation.

Figure S24. Representative dose-response curves for the treatment of MCF10A cells with (A) **1**, (B) **2**, (C) **3** or (D) **4** after 72 h incubation.

Table S8. IC_{50} values of **1-4** against BEAS-2B cells. [a] Determined after 72 h incubation (mean of three independent experiments ± SD).

Figure S25. Representative dose-response curves for the treatment of HMLER-shEcad mammospheres with copper(II) complexes **1-4** after 5 days incubation.

Figure S26. The amount of copper (in terms of ng of Cu/ million cells) present in HMLER-shEcad cells treated with **1-4** (3 μM for 24 h).

Figure S27. Copper content (ng of Cu/ 10^6 cells) in various cellular components upon treatment of HMLER-shEcad cells with **4** (3 μM for 24 h).

Figure S28. Immunoblotting analysis of proteins related to the JNK/p38 and apoptosis pathways. Protein expression in HMLER-shEcad cells following treatment with **4** (5, 10, and 20 μM) after 72 h incubation.

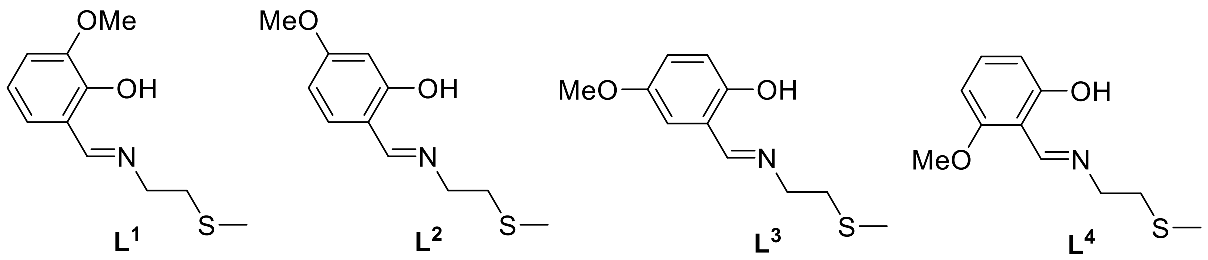

Figure S1. Chemical structures of the vanillin Schiff base ligands **L¹-L⁴**.

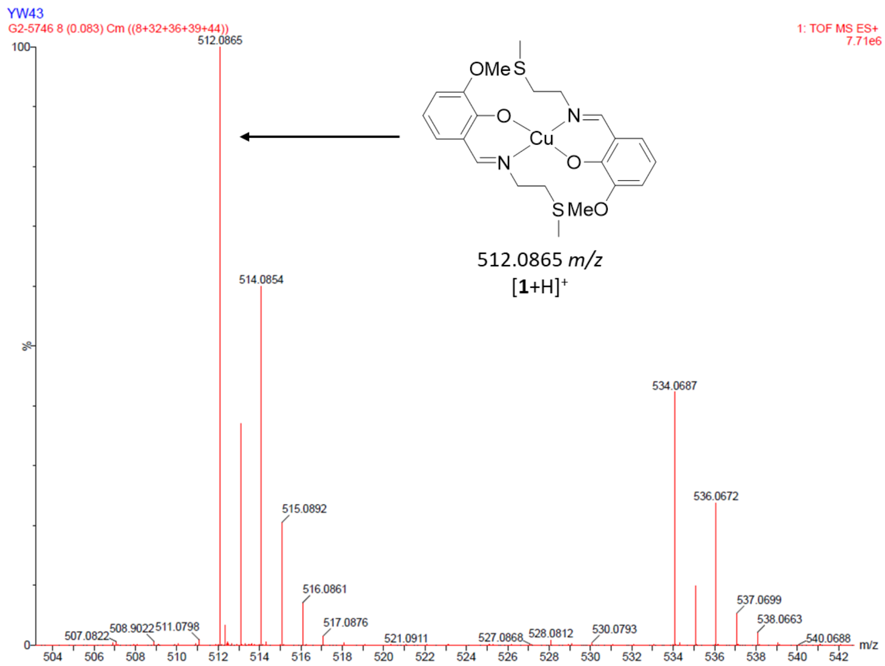

Figure S2. High resolution ESI mass spectrum (positive mode) of **1**.

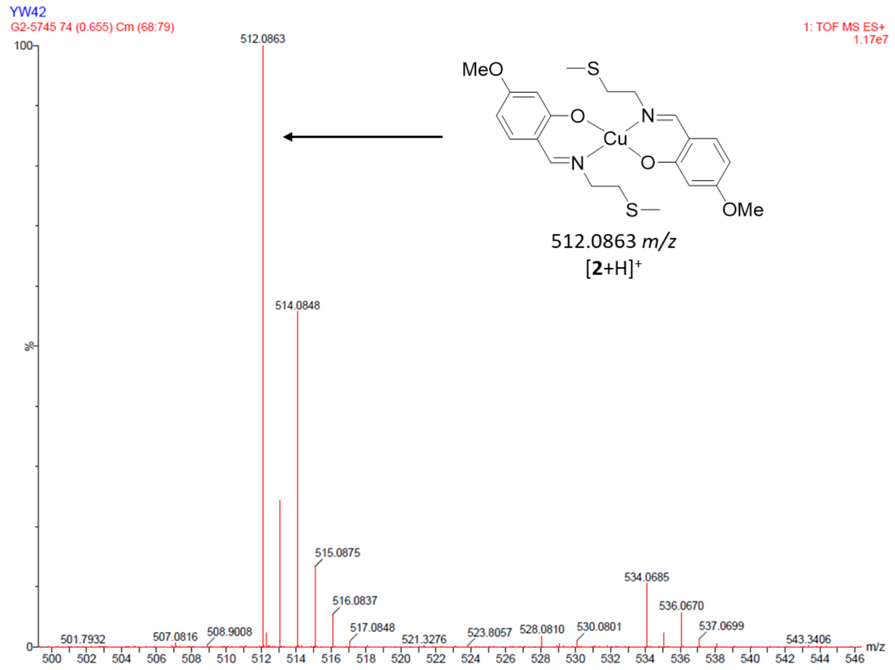

Figure S3. High resolution ESI mass spectrum (positive mode) of **2**.

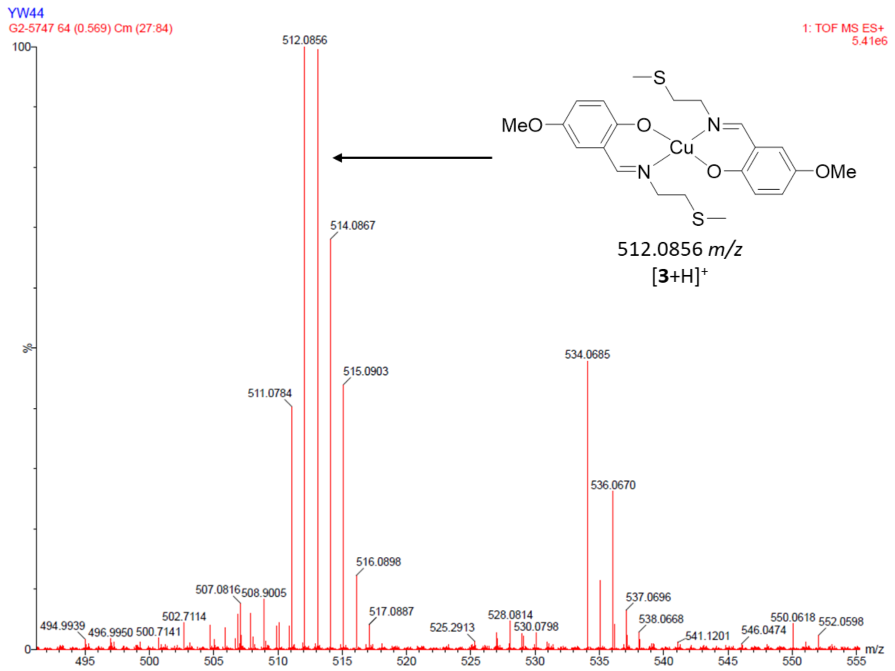

Figure S4. High resolution ESI mass spectrum (positive mode) of **3**.

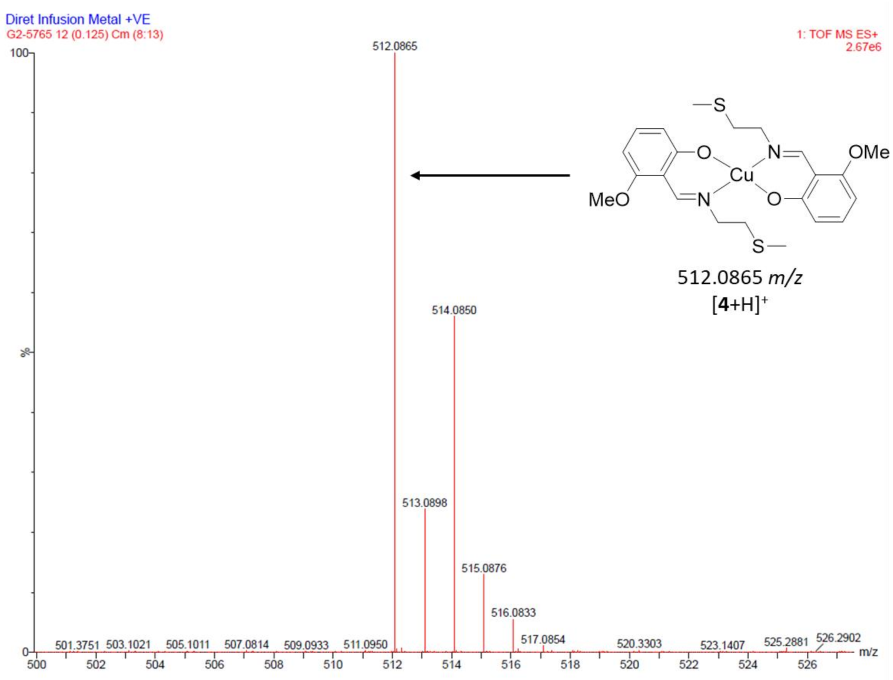

Figure S5. High resolution ESI mass spectrum (positive mode) of **4**.

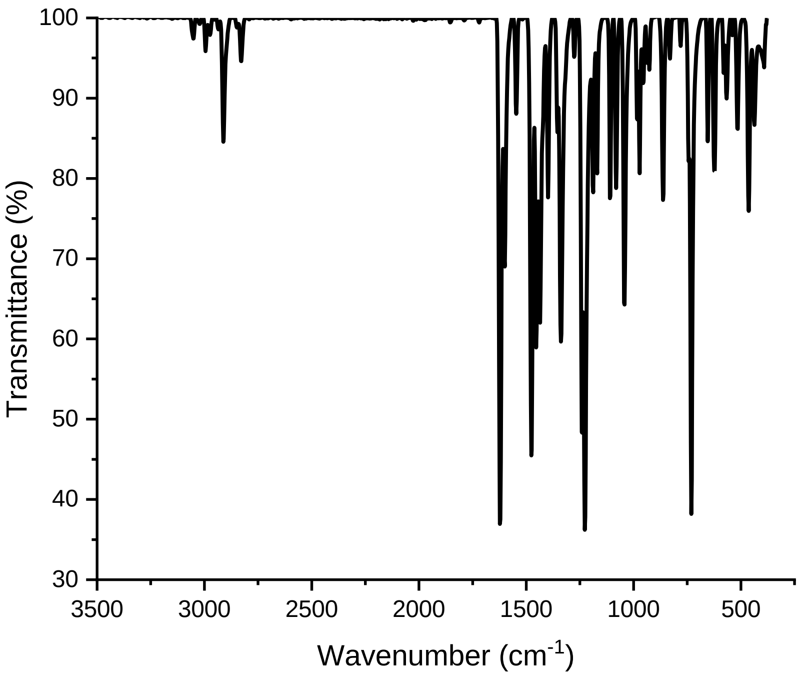

Figure S6. ATR-FTIR spectrum of **1** in the solid form.

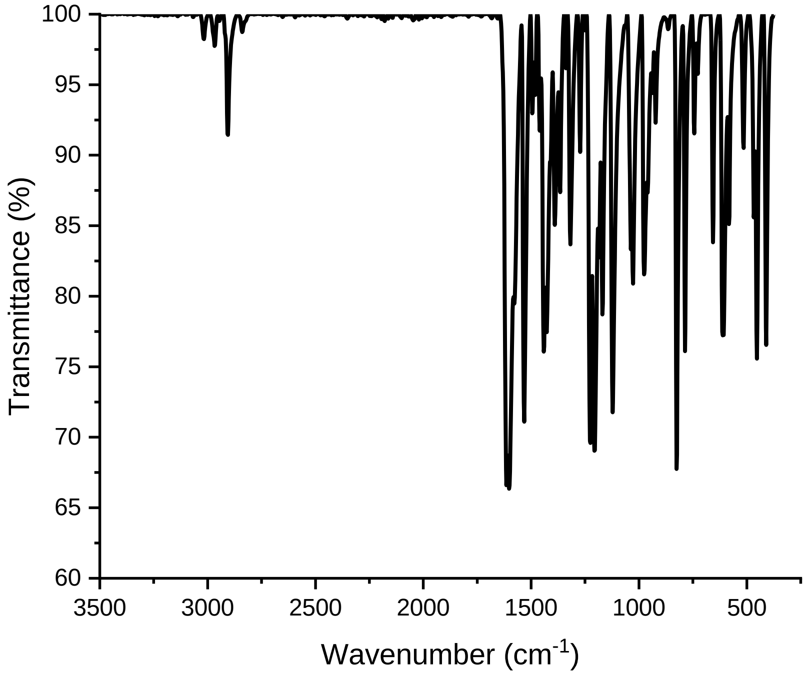

Figure S7. ATR-FTIR spectrum of **2** in the solid form.

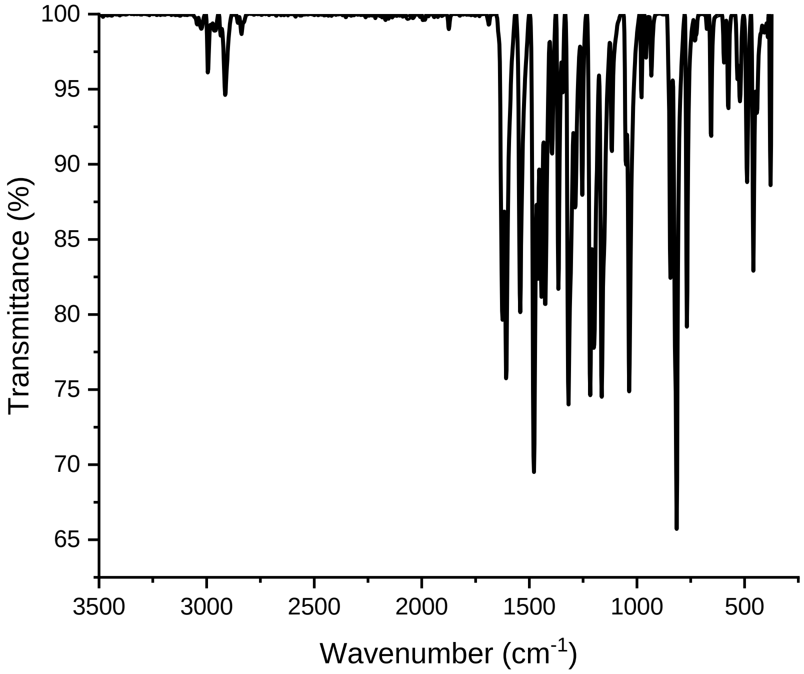

Figure S8. ATR-FTIR spectrum of **3** in the solid form.

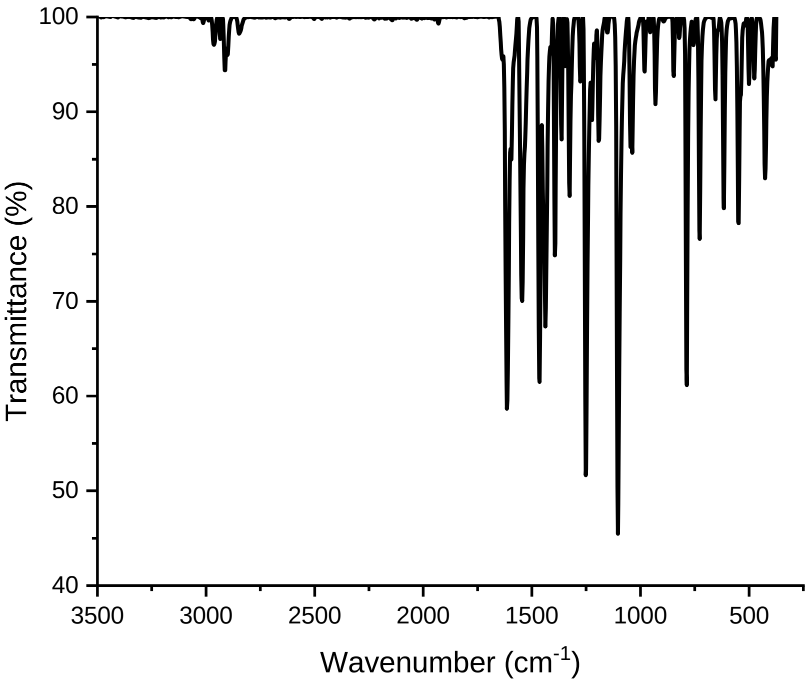

Figure S9. ATR-FTIR spectrum of **4** in the solid form.

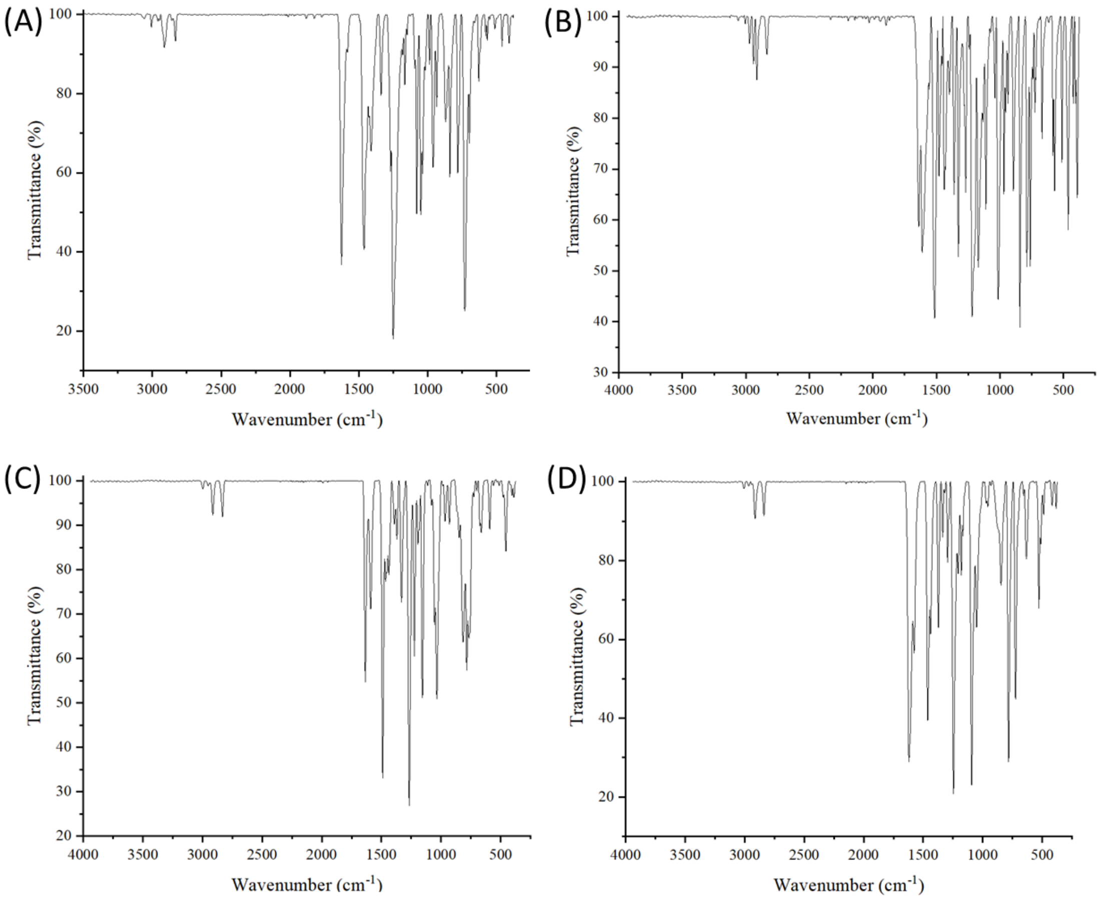

Figure S10. ATR-FTIR spectrum of (A) $\mathbf{L^1}$, (B) $\mathbf{L^2}$, (C) $\mathbf{L^3}$, and (D) $\mathbf{L^4}$ in the liquid form.

Table S1. Crystallographic data for copper(II) complexes **1** and **2**.

**Metal complex**	**1**	**2**
CCDC No.	2426496	2426497
formula	$C_{22}H_{28}CuN_2O_4S_2$	$C_{22}H_{28}CuN_2O_4S_2$
Fw	512.12	512.12
Crystal system	triclinic	monoclinic
Space group	P-1	$P2_1$
a, Å	4.6804(3)	10.3017(2)
b, Å	10.9543(6)	7.9600(2)
c, Å	11.0945(6)	14.2343(3)
α, deg.	92.099(2)	90
β, deg.	99.023(2)	96.7810(10)
γ, deg.	98.038(2)	90
V, Å^3	555.26(6)	1159.07(4)
Z	1	2
D_{calcd}, Mg/m^3	1.532	1.467
2θ / deg.	8.084 to 144.382	8.644 to 144.204
Reflections collected	7480	13950
Independent reflections	2140(0.0475, 0.0482)	4399(0.0307, 0.0300)
Goodness-of-fit on F^2	1.063	1.078
R_1, wR_2 [$I \geq 2\sigma(I)$]	0.0385, 0.1078	0.0469, 0.1284
R_1, wR_2 [all data]	0.0395, 0.1090	0.0475, 0.1290

The copper(II) complex **1** has been generated by symmetry, symmetry operation -*x*+2, -*y*+1, -*z*+1.

Table S2. Crystallographic data for copper(II) complexes **3** and **4**.

**Metal complex**	**3**	**4**
CCDC No.	2426498	2426499
formula	$C_{22}H_{28}CuN_2O_4S_2$	$C_{22}H_{28}CuN_2O_4S_2$
Fw	512.12	512.12
Crystal system	triclinic	monoclinic
Space group	P-1	$P2_1/c$
a, Å	5.2457(2)	5.5443(3)
b, Å	9.6211(3)	18.5685(10)
c, Å	11.3998(4)	10.8973(5)
α, deg.	78.9350(10)	90
β, deg.	81.4650(10)	90.913(2)
γ, deg.	89.3510(10)	90
V, Å^3	558.31(3)	1121.73(10)
Z	1	2
D_{calcd}, Mg/m^3	1.523	1.516
2θ / deg.	9.368 to 144.678	9.41 to 144.718
Reflections collected	8169	13111
Independent reflections	2188(0.0577, 0.0519)	2193(0.0485, 0.0346)
Goodness-of-fit on F^2	1.064	1.137
R_1, wR_2 [$I \geq 2\sigma(I)$]	0.0533, 0.1452	0.0313, 0.0985
R_1, wR_2 [all data]	0.0590, 0.1479	0.0376, 0.0998

Half of copper(II) complex **3** has been generated by symmetry, symmetry operation -*x*+2, -*y*+1, -*z*+1. Half of copper(II) complex **4** has been generated by symmetry, symmetry operation -*x*, -*y*+1, -*z*+1.

Table S3. Selected bond lengths (Å) and angles (°) for copper(II) complex **1**.

Cu(1)-O(1)	1.8994(13)	O(1)-Cu(1)-O(1^1)	180.00(7)
Cu(1)-O(1^1)	1.8994(13)	O(1^1)-Cu(1)-N(1^1)	91.61(6)
Cu(1)-N(1)	2.0057(15)	O(1)-Cu(1)-N(1^1)	88.39(6)
Cu(1)-N(1^1)	2.0057(15)	O(1)-Cu(1)-N(1)	91.60(6)
		O(1^1)-Cu(1)-N(1)	88.40(6)
		N(1)-Cu(1)-N(1^1)	180.0

Table S4. Selected bond lengths (Å) and angles (°) for copper(II) complex **2**.

Cu(1)-O(1)	1.892 (4)	O(1)-Cu(1)-O(3)	175.62 (19)
Cu(1)-O(3)	1.894 (4)	O(1)-Cu(1)-N(1)	90.37 (17)
Cu(1)-N(1)	2.024 (4)	O(1)-Cu(1)-N(2)	88.59 (17)
Cu(1)-N(2)	2.000 (4)	O(3)-Cu(1)-N(1)	88.03 (16)
		O(3)-Cu(1)-N(2)	91.99 (16)
		N(2)-Cu(1)-N(1)	179.58 (19)

Table S5. Selected bond lengths (Å) and angles (°) for copper(II) complex **3**.

Cu(1)- O(1^1)	1.890 (2)	O(1^1)-Cu(1)-O(1)	180.0
Cu(1)-O1	1.890 (2)	O(1)-Cu(1)-N(1)	91.72 (9)
Cu(1)- N(1^1)	2.004 (2)	O(1^1)-Cu(1)-N(1)	88.28 (9)
Cu(1)-N(1)	2.004 (2)	O(1^1)-Cu(1)-N(1^1)	91.72 (9)
		O(1)-Cu(1)-N(1^1)	88.28 (9)
		N(1^1)-Cu(1)-N(1)	180.0

Table S6. Selected bond lengths (Å) and angles (°) for copper(II) complex **4**.

Cu(1)-O(1)	1.8829 (12)	O(1)-Cu(1)-O(1^1)	180.0
Cu(1)-O(1^1)	1.8829 (12)	O(1)-Cu(1)-N(1^1)	88.62 (5)
Cu(1)-N(1)	2.0065 (13)	O(1^1)-Cu(1)-N(1^1)	91.38 (5)
Cu(1)-N(1^1)	2.0065 (13)	O(1)-Cu(1)-N(1)	91.38 (6)
		O(1^1)-Cu(1)-N(1)	88.62 (5)
		N(1)-Cu(1)-N(1^1)	180.0

Table S7. Experimentally determined LogP values for the copper(II) complexes **1-4**.

Copper(II) complex	LogP value
**1**	0.75 ± 0.01
**2**	0.37 ± 0.04
**3**	0.53 ± 0.01
**4**	0.34 ± 0.003

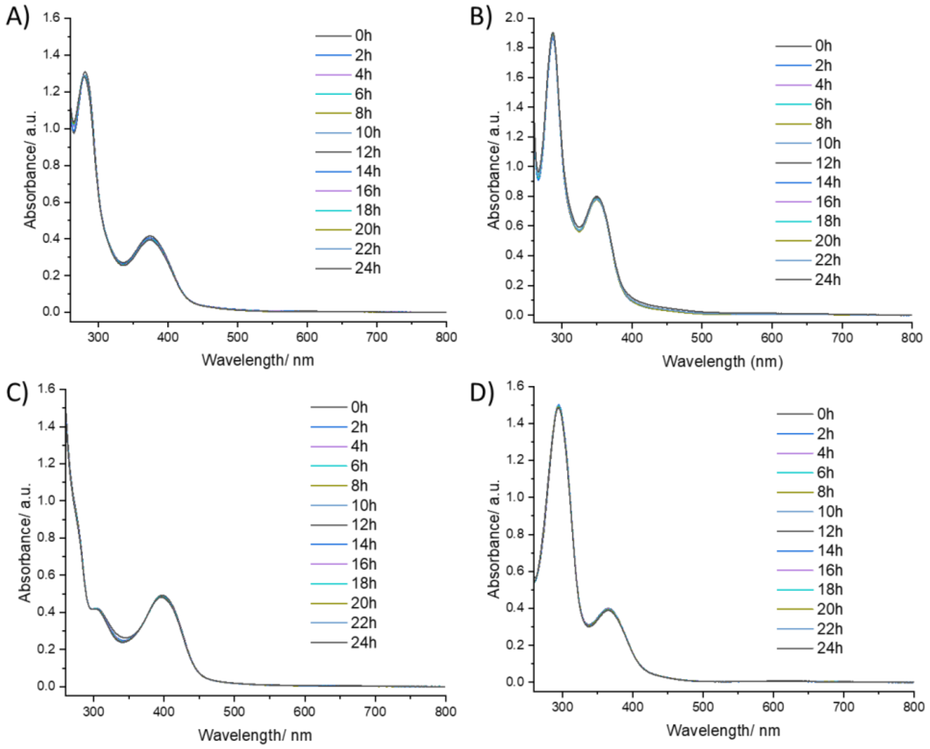

Figure S11. UV-Vis spectra of (A) **1**, (B) **2**, (C) **3**, and (D) **4** (all 50 μM) in DMSO over the course of 24 h at 37 °C.

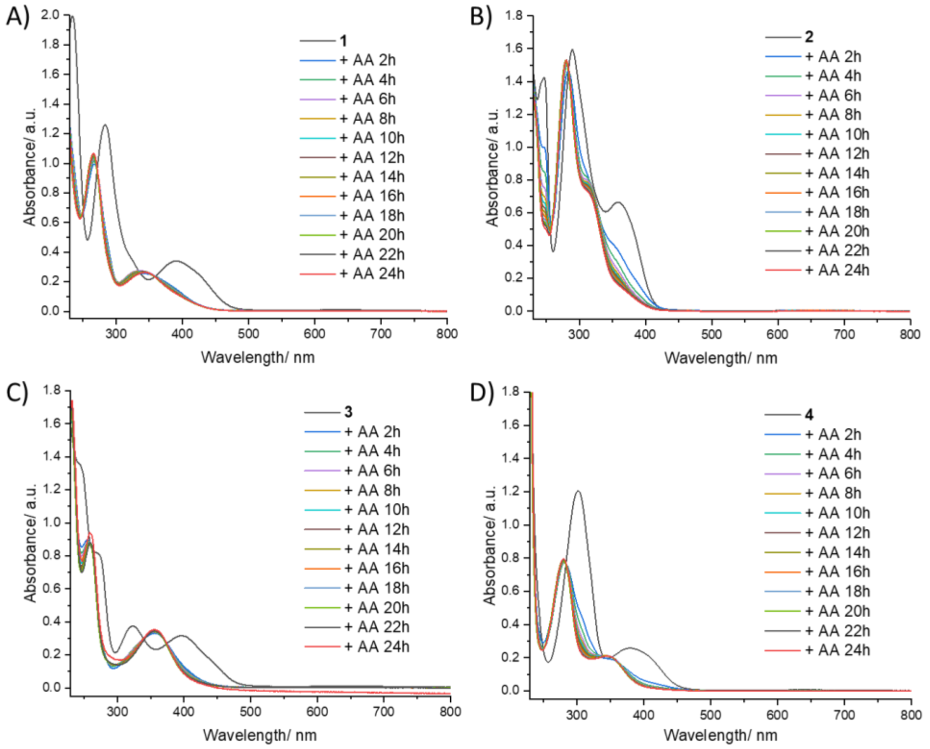

Figure S12. UV-Vis spectra of (A) **1**, (B) **2**, (C) **3**, and (D) **4** (all 50 μM) in PBS:DMSO (200:1) in the presence of ascorbic acid (10 equivalences) over the course of 24 h at 37 °C.

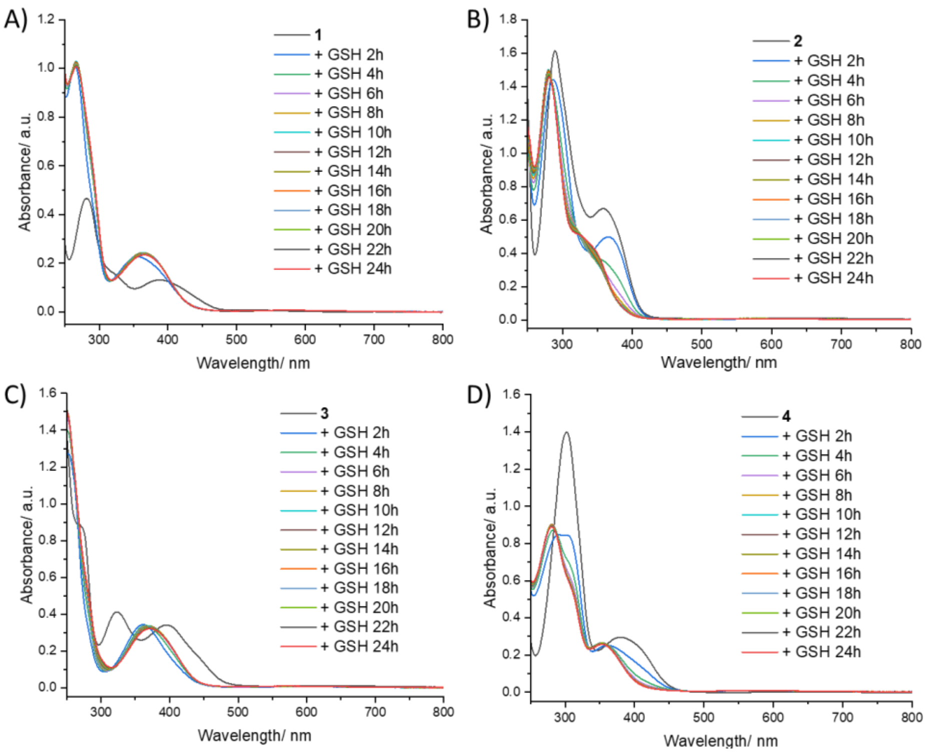

Figure S13. UV-Vis spectra of (A) **1**, (B) **2**, (C) **3**, and (D) **4** (all 50 μM) in PBS:DMSO (200:1) in the presence of glutathione (10 equivalences) over the course of 24 h at 37 °C.

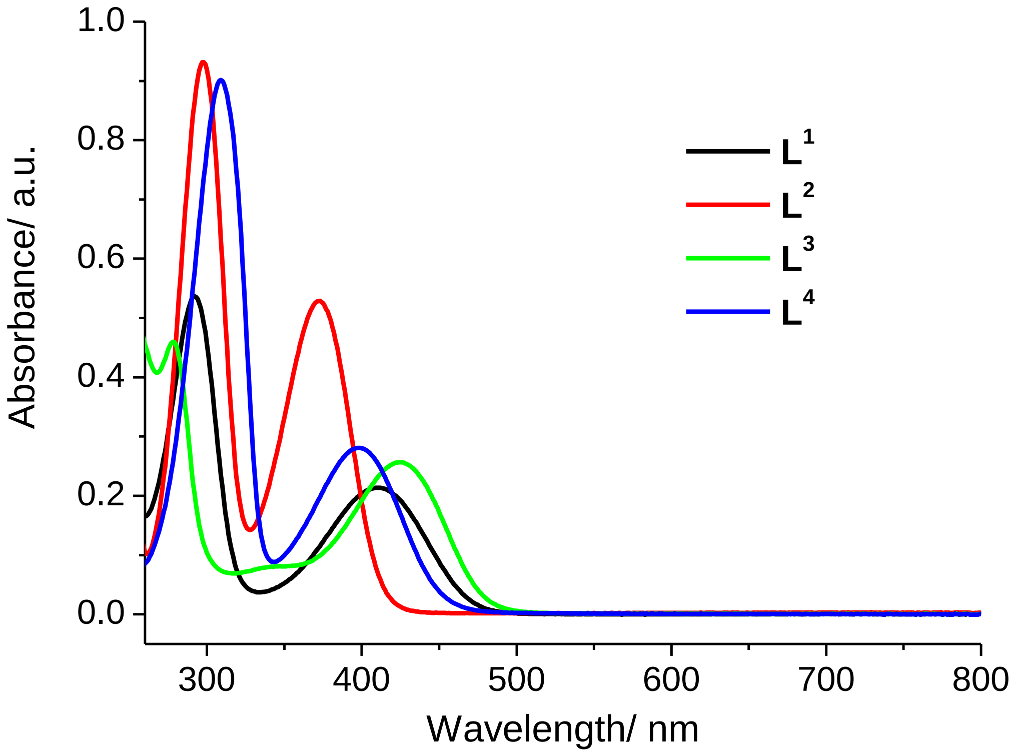

Figure S14. UV-Vis spectra of **L^1**, **L^2**, **L^3**, and **L^4** (all 50 μM) in PBS:DMSO (200:1) at 37 °C.

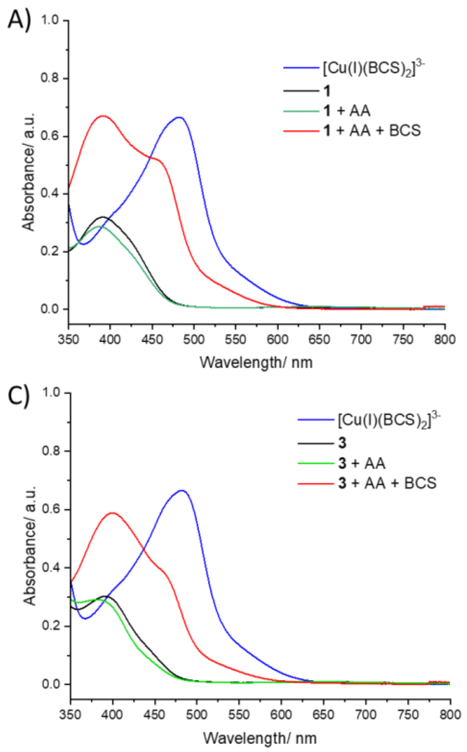

Figure S15. UV-Vis spectra of (A) **1**, (B) **2**, (C) **3**, and (D) **4** (all 50 μM) in PBS:DMSO (200:1) in the presence of ascorbic acid (10 equivalences) and bathocuproine disulfonate, BCS (2 equivalences) at 37 ^{o}C.

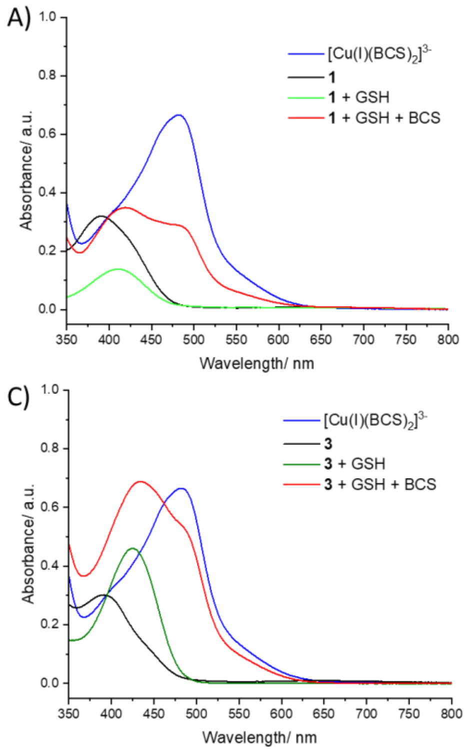

Figure S16. UV-Vis spectra of (A) **1**, (B) **2**, (C) **3**, and (D) **4** (all 50 μM) in PBS:DMSO (200:1) in the presence of glutathione (10 equivalences) and bathocuproine disulfonate, BCS (2 equivalences) at 37 ^{o}C.

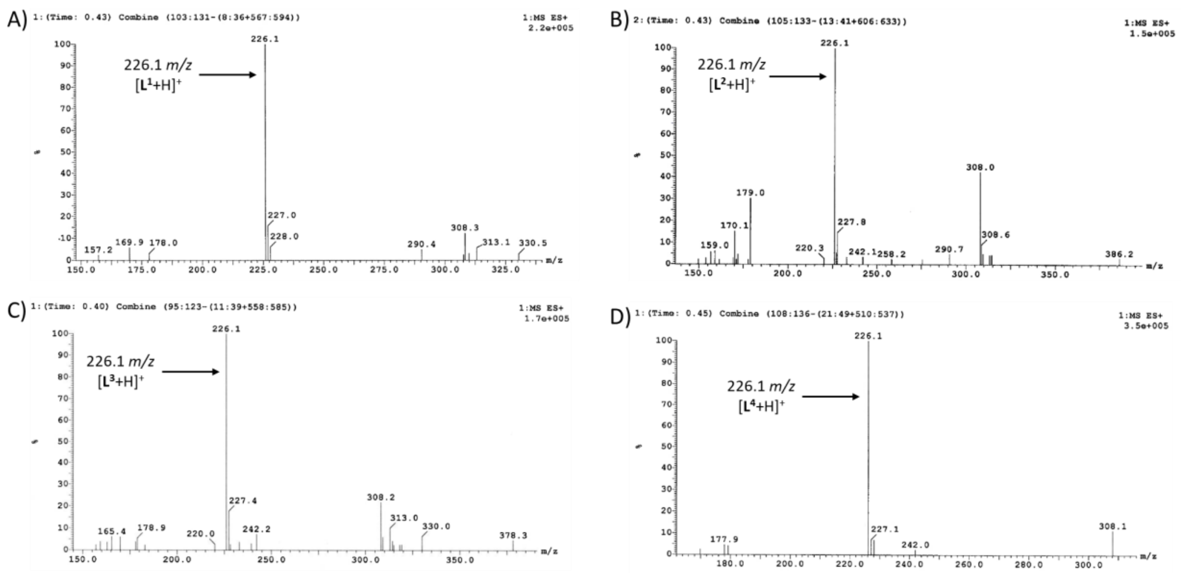

Figure S17. ESI mass spectra (positive mode) of (A) **1**, (B) **2**, (C) **3**, and (D) **4** (all 500 μM) in H_2O:DMSO (10:1) in the presence of glutathione (10 equivalences) after incubation for 24 h at 37 °C.

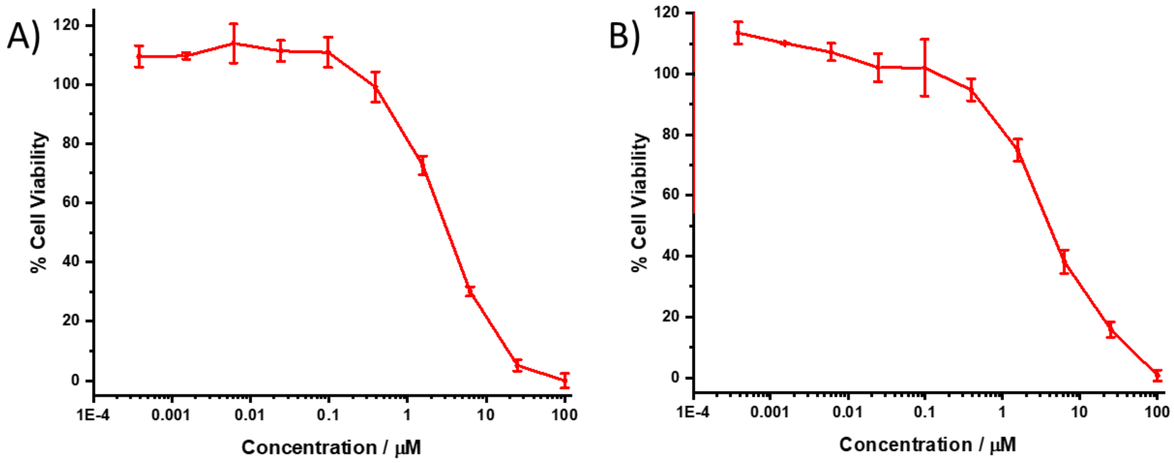

Figure S18. Representative dose-response curves for the treatment of (A) HMLER and (B) HMLER-shEcad cells with **1** after 72 h incubation.

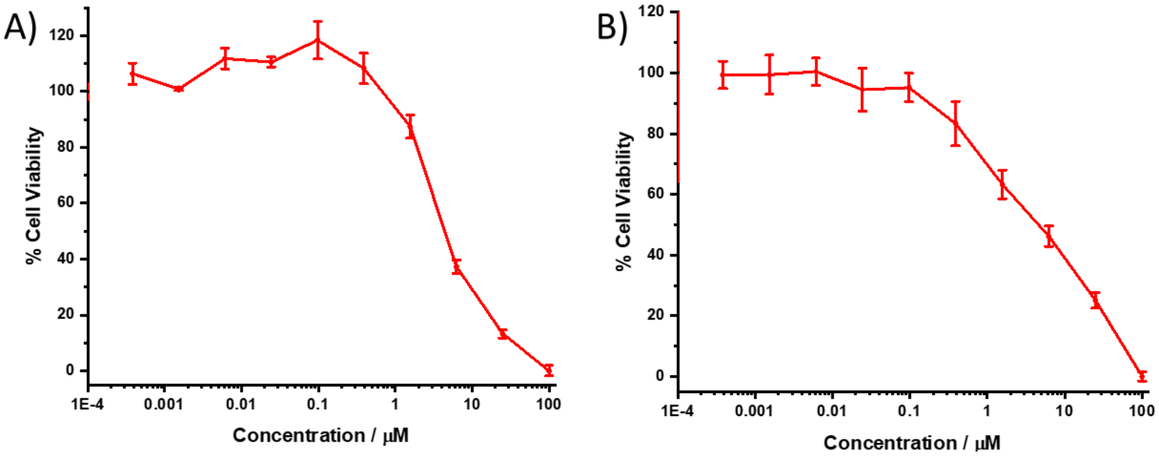

Figure S19. Representative dose-response curves for the treatment of (A) HMLER and (B) HMLER-shEcad cells with **2** after 72 h incubation.

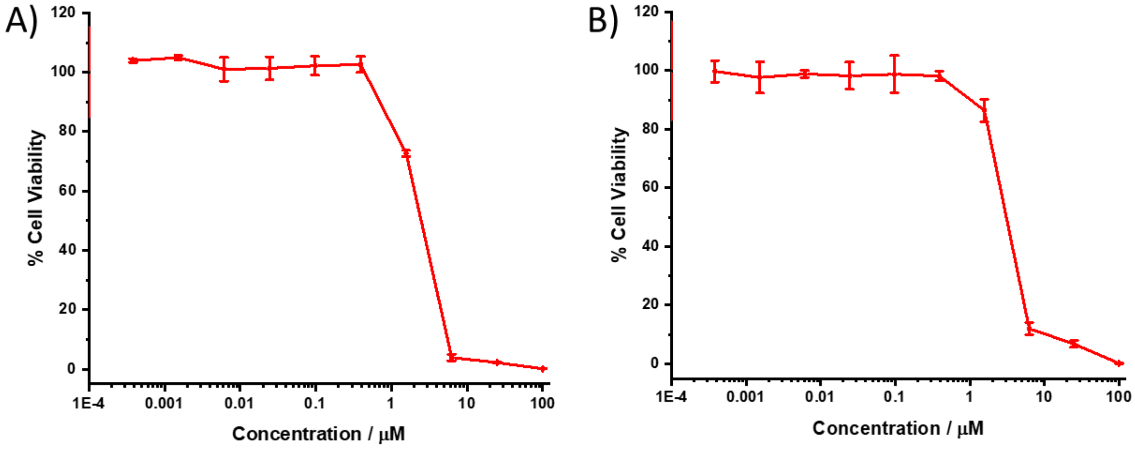

Figure S20. Representative dose-response curves for the treatment of (A) HMLER and (B) HMLER-shEcad cells with **3** after 72 h incubation.

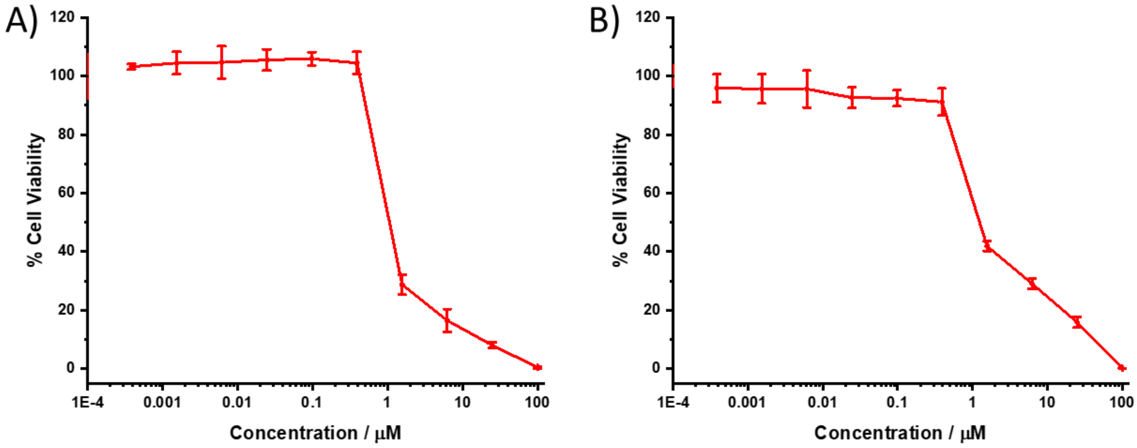

Figure S21. Representative dose-response curves for the treatment of (A) HMLER and (B) HMLER-shEcad cells with **4** after 72 h incubation.

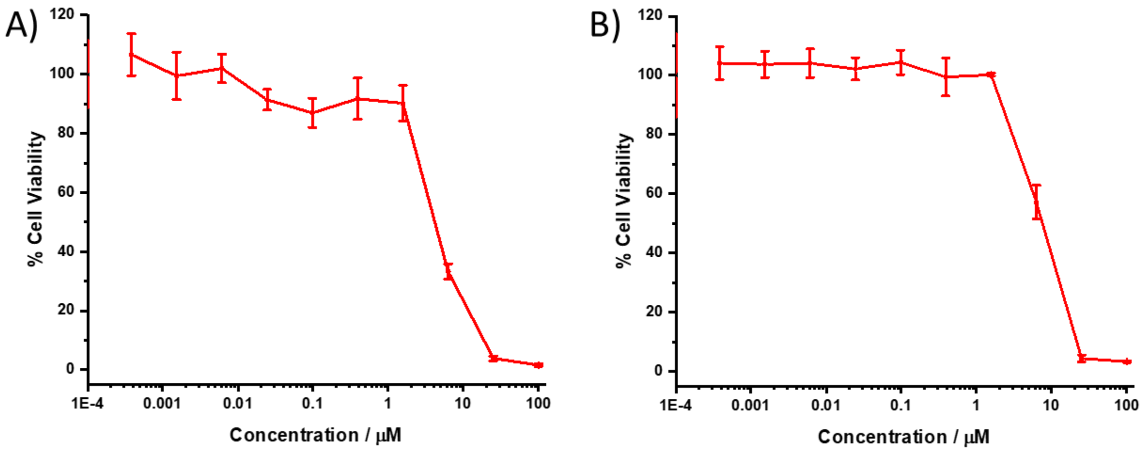

Figure S22. Representative dose-response curves for the treatment of (A) HMLER and (B) HMLER-shEcad cells with **L**^{4} after 72 h incubation.

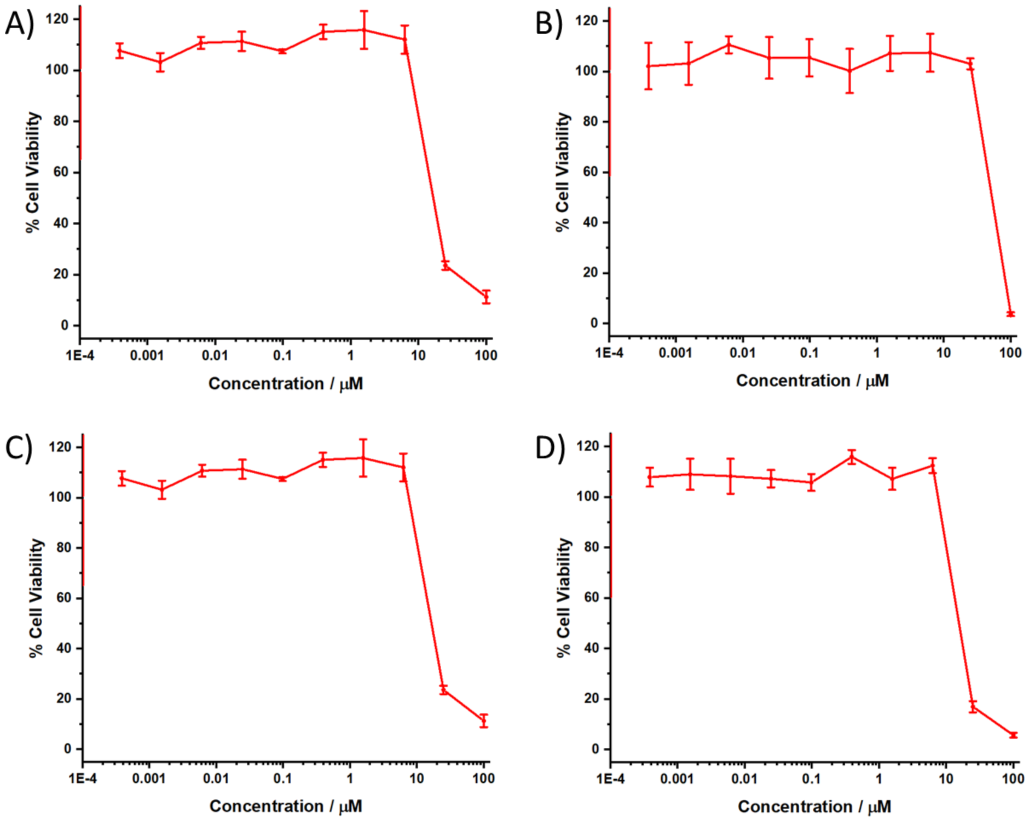

Figure S23. Representative dose-response curves for the treatment of BEAS-2B cells with (A) **1**, (B) **2**, (C) **3** or (D) **4** after 72 h incubation.

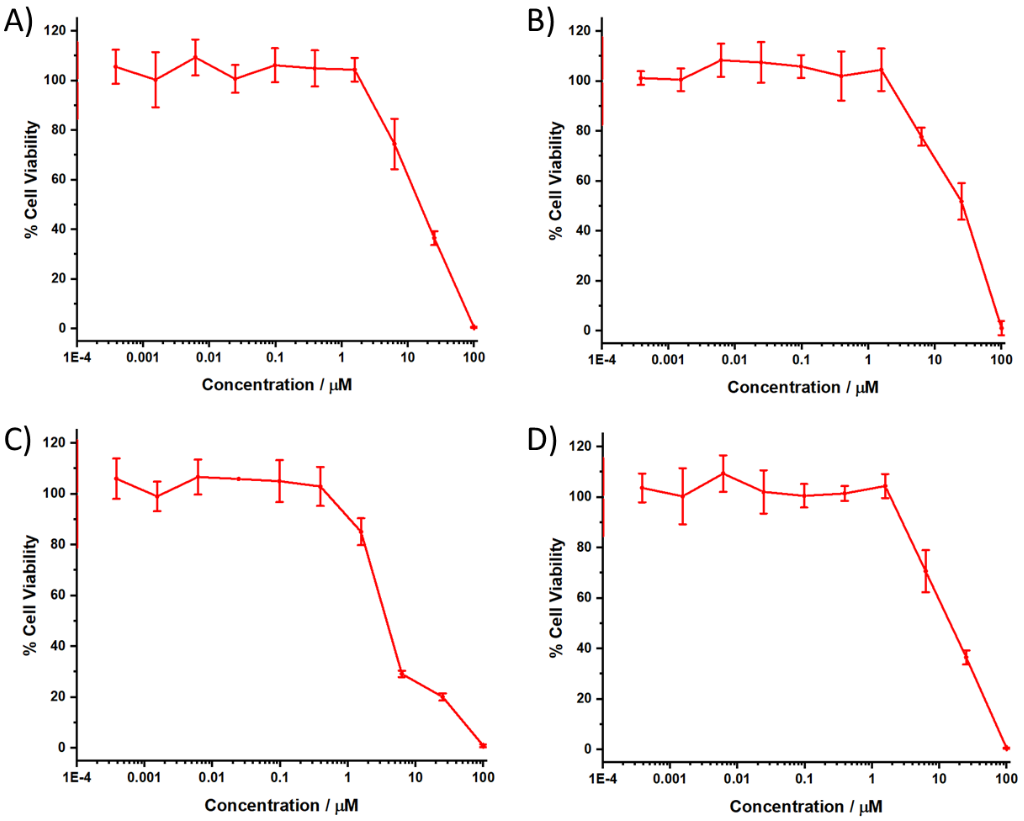

Figure S24. Representative dose-response curves for the treatment of MCF10A cells with (A) **1**, (B) **2**, (C) **3** or (D) **4** after 72 h incubation.

Table S8. IC_{50} values of **1-4** against BEAS-2B and MCF10A cells. [a] Determined after 72 h incubation (mean of three independent experiments ± SD).

Test compound	BEAS-2B [µM][a]	MCF10A [µM][a]
**1**	16.07 ± 0.45	15.27 ± 0.98
**2**	50.69 ± 0.54	25.66 ± 3.43
**3**	16.35 ± 0.64	3.71 ± 0.03
**4**	15.17 ± 0.21	13.62 ± 3.20

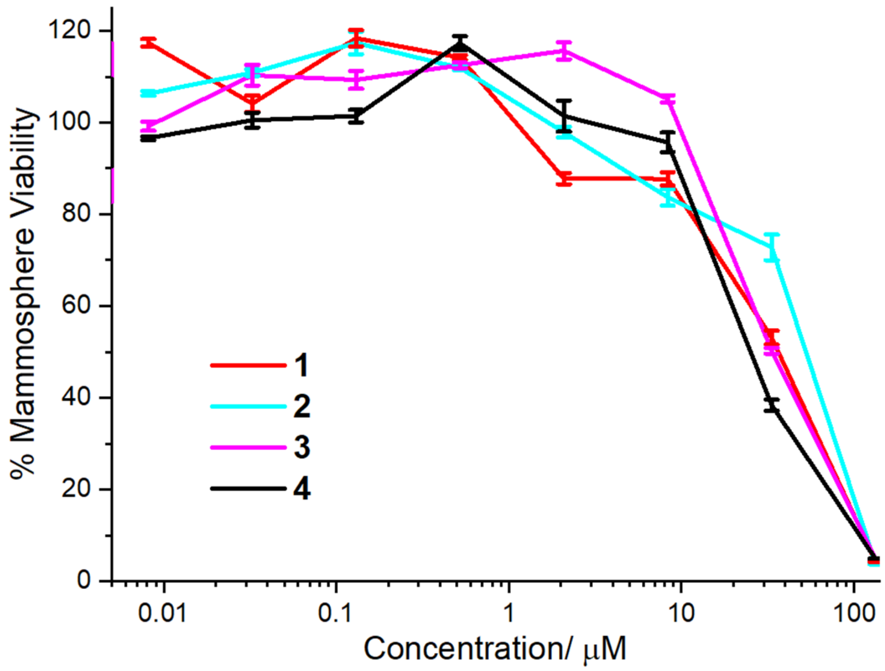

Figure S25. Representative dose-response curves for the treatment of HMLER-shEcad mammospheres with copper(II) complexes **1-4** after 5 days incubation.

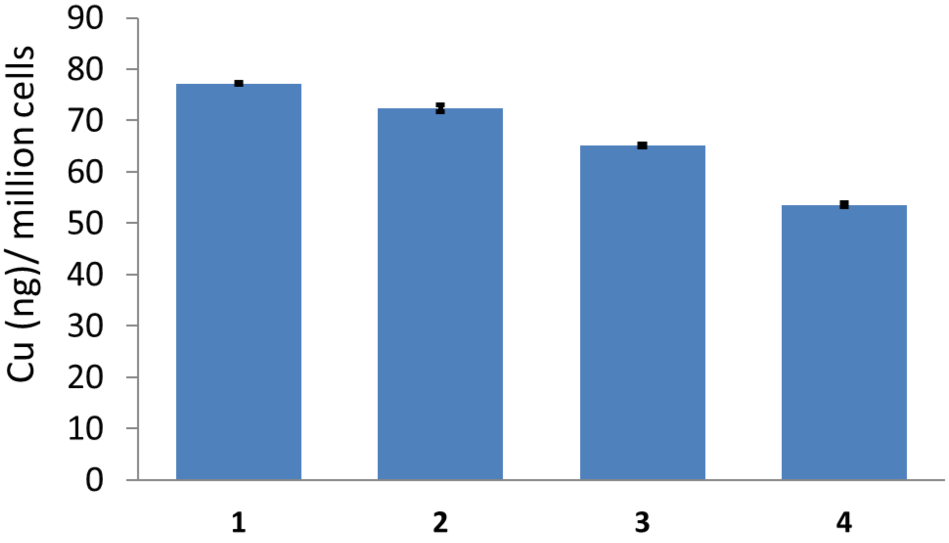

Figure S26. The amount of copper (in terms of ng of Cu/ million cells) present in HMLER-shEcad cells treated with **1-4** (3 µM for 24 h).

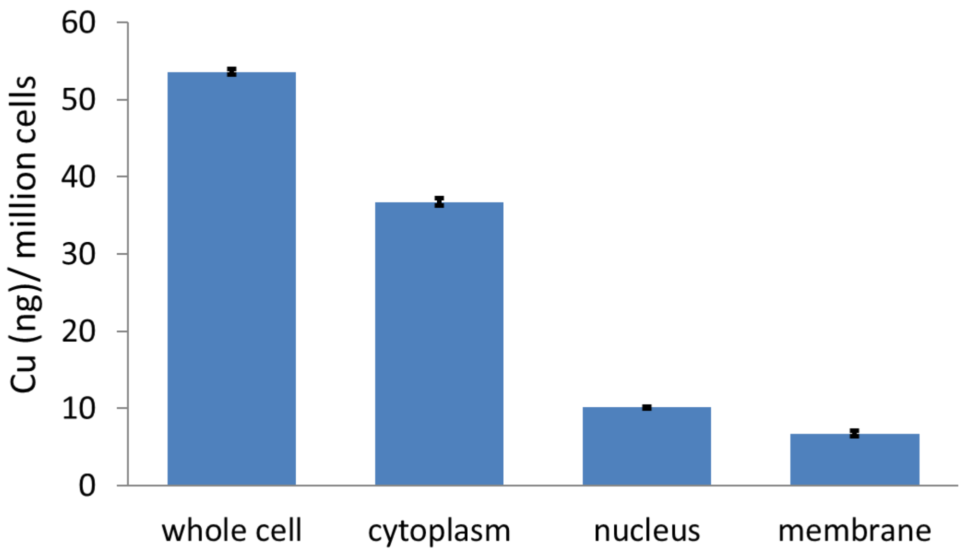

Figure S27. Copper content (ng of Cu/ 10^6 cells) in various cellular components upon treatment of HMLER-shEcad cells with **4** (3 µM for 24 h).

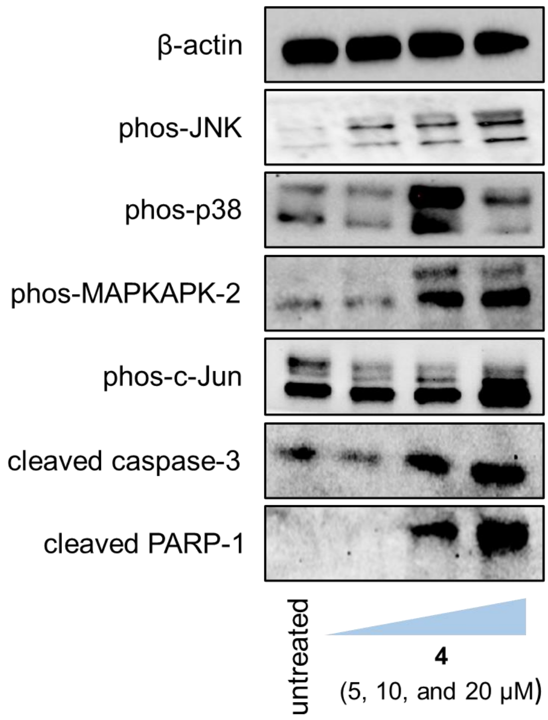

Figure S28. Immunoblotting analysis of proteins related to the JNK/p38 and apoptosis pathways. Protein expression in HMLER-shEcad cells following treatment with **4** (5, 10, and 20 µM) after 72 h incubation.
